# Principles and framework for assessing the risk of bias for studies included in comparative quantitative environmental systematic reviews

**DOI:** 10.1186/s13750-022-00264-0

**Published:** 2022-03-29

**Authors:** Geoff Frampton, Paul Whaley, Micah Bennett, Gary Bilotta, Jean-Lou C. M. Dorne, Jacqualyn Eales, Katy James, Christian Kohl, Magnus Land, Barbara Livoreil, David Makowski, Evans Muchiri, Gillian Petrokofsky, Nicola Randall, Kate Schofield

**Affiliations:** 1 Southampton Health Technology Assessments Centre (SHTAC), Faculty of Medicine, University of Southampton, Southampton, UK.; 2 Lancaster Environment Centre, Lancaster University, Lancaster, UK.; 3 Evidence-Based Toxicology Collaboration at Johns Hopkins Bloomberg School of Public Health, Baltimore, USA.; 4 U.S. Environmental Protection Agency, Region 5, Chicago, IL 60604, USA.; 5 School of Environment and Technology, University of Brighton, Brighton, UK.; 6 Scientific Committee and Emerging Risks Unit, European Food Safety Authority, Via Carlo Magno 1A, 43121 Parma, Italy.; 7 European Centre for Environment and Human Health, College of Medicine and Health, University of Exeter, Knowledge Spa, Truro TR1 3HD, UK.; 8 Centre for Evidence-Based Agriculture, Harper Adams University, Newport, Shropshire TF10 8NB, UK.; 9 Institute for Biosafety in Plant Biotechnology (SB), Julius Kühn Institute (JKI) - Federal Research Centre for Cultivated Plants, Berlin, Germany.; 10 Formas, Box 1206, 111 82 Stockholm, Sweden.; 11 Freelance Consultant, Coopaname, France.; 12 UMR518, University Paris-Saclay, INRAE, AgroParistech, 16 rue Claude Bernard, 75231 Paris, France.; 13 Centre for Anthropological Research, University of Johannesburg, Johannesburg, South Africa.; 14 Oxford Long-Term Ecology Lab, Department of Zoology, University of Oxford, Oxford, UK.; 15 Centre for Evidence-Based Agriculture, Harper Adams University, Newport, Shropshire TF10 8NB, UK.; 16 Office of Research and Development, U.S. Environmental Protection Agency, Washington, DC 20460, USA.

**Keywords:** Validity, Bias, Risk of bias, Systematic error, Internal validity, External validity, Quality assessment, Critical appraisal, Blinding

## Abstract

The internal validity of conclusions about effectiveness or impact in systematic reviews, and of decisions based on them, depends on risk of bias assessments being conducted appropriately. However, a random sample of 50 recently-published articles claiming to be quantitative environmental systematic reviews found 64% did not include any risk of bias assessment, whilst nearly all that did omitted key sources of bias. Other limitations included lack of transparency, conflation of quality constructs, and incomplete application of risk of bias assessments to the data synthesis. This paper addresses deficiencies in risk of bias assessments by highlighting core principles that are required for risk of bias assessments to be fit-for-purpose, and presenting a framework based on these principles to guide review teams on conducting risk of bias assessments appropriately and consistently. The core principles require that risk of bias assessments be Focused, Extensive, Applied and Transparent (FEAT). These principles support risk of bias assessments, appraisal of risk of bias tools, and the development of new tools. The framework follows a Plan-Conduct-Apply-Report approach covering all stages of risk of bias assessment. The scope of this paper is comparative quantitative environmental systematic reviews which address PICO or PECO-type questions including, but not limited to, topic areas such as environmental management, conservation, ecosystem restoration, and analyses of environmental interventions, exposures, impacts and risks.

## Introduction: assessing risk of bias in quantitative environmental systematic reviews

Quantitative systematic reviews (i.e. those which synthesise numerical data from studies) require an assessment of the risk of bias for each of the individual studies included in the review. Any issues with bias in the individual studies can then be considered when formulating the review conclusions. The assessment of the risk of bias takes place at the “risk of bias” stage in the systematic review process, often also known as the “critical appraisal” or “quality assessment” stage [1–5]. Risk of bias assessment is a key defining feature of quantitative systematic reviews that is often absent from traditional narrative reviews. Risk of bias assessment is arguably one of the most challenging stages of an environmental systematic review, for several reasons (Box 1).

### Rationale for this paper

Systematic reviews are conducted widely across a range of disciplines, and for some areas such as human health and the social sciences detailed guidance and standards are available [1, 3, 5]. More recently, the Collaboration for Environmental Evidence (CEE), a not-for-profit organisation promoting best practice in evidence synthesis, has developed Guidelines and Standards for Evidence Synthesis of Environmental Management Topics (updated April 2018) [4]. However, there is evidence suggesting that the risk of bias stage of environmental systematic reviews is not always conducted to the best possible standards. For example, in a random sample of environmental articles published between 2018 and 2020 that claimed in their title or abstract to be systematic reviews, 64% of the reviews had not conducted risk of bias assessment at all (Additional file 1).

Systematic reviews that are registered with CEE are expected to follow CEE’s Guidelines and Standards [4] and are published in the journal Environmental Evidence. They are likely to be among the most rigorous of environmental systematic reviews. For example, all the CEE systematic reviews in the random sample had conducted risk of bias assessment, compared to only 26% of the non-CEE systematic reviews (Additional file 1). However, CEE has recognised that the current Guidelines and Standards [4] do not address all of the challenges faced by review teams in relation to assessing the risk of bias (Box 1). The authors of the present paper were requested by CEE to develop more detailed guidance on risk of bias assessment, based on an evaluation of current approaches employed in CEE systematic reviews and consideration of guidance available in other disciplines. Despite the generally high evidence synthesis standards reflected in CEE systematic reviews, we found that in a sample of the 10 most recently published CEE systematic reviews (up to June 2021) [6–15], each review developed review-specific bias or quality assessment instruments with limited consistency across reviews, varying degrees of detail and transparency, and occasional omission of key classes of bias (Additional file 2)—all of which suggest room for improvement in risk of bias guidance [4].

This paper aims to provide guidance for review teams conducting risk of bias assessments within comparative quantitative environmental systematic reviews, specifically those that focus on PICO or PECO-type questions (see “Scope of the paper” section below), addressing where possible the challenges listed in Box 1. To ensure that risk of bias assessments are as consistent as possible and fit-for-purpose we highlight four core principles that risk of bias assessments must meet (FEAT: assessments must be Focused, Extensive, Applied and Transparent). A comprehensive Plan-Conduct-Apply-Report framework based on these principles is provided to guide review teams at all stages of the planning, conduct, application and reporting of risk of bias assessment, from the development of the systematic review protocol to the presentation of the completed assessment in the final systematic review report. The FEAT principles provide a rational basis for structuring risk of bias assessments and can also be used to assess the fitness-for-purpose of risk of bias tools or assist in the modification of existing tools (where allowed by copyright terms) or development of new tools where necessary.

### Scope of the paper

#### Types of systematic review questions and topics covered

This paper focuses on comparative quantitative systematic reviews, that is, those which synthesise numerical data from studies, through narrative syntheses and/or meta-analyses. It is specifically applicable to reviews addressing questions with “PICO” or “PECO” structure, where the Population(s), Intervention(s) or Exposure(s), Comparator(s) and Outcome(s) of interest are explicitly specified or deducible. The process of risk of bias assessment is the same for studies addressing exposure (PECO) and intervention (PICO) questions [16]. For simplicity in this paper we use the term “exposure” to cover both exposures (e.g. naturally occurring substances or events) and interventions (e.g. intentionally applied anthropogenic substances or intentionally implemented events). This paper does not cover systematic reviews that employ purely qualitative data synthesis of qualitative studies (e.g. realist synthesis), since qualitative systematic reviews employ a different paradigm for critically assessing individual studies [17].

The subject scope of this paper is broad, and applicable to any environmental topics including, but not limited to, environmental management, conservation, ecosystem restoration, and analyses of environmental interventions, exposures, impacts and risks.

#### Types of validity covered

Validity refers to the extent of *systematic error* (see “Internal validity (systematic error) and precision (random error) of studies” section below). Two types of validity are relevant to the individual studies included in a systematic review.

Internal validity describes the extent of systematic error inherent to an individual study and reflects the extent to which the study’s methods can provide an unbiased result. Internal validity is assessed as the *risk of bias*.

External validity describes the extent of systematic error in applying the results of a study to answer a specific question, which may also be referred to as “generalisability”, “applicability” or “directness” [18, 19].

The focus of this paper is specifically on internal validity, i.e. the risk of bias. This focus relates to the challenges outlined in Box 1. Both the internal and external validity of studies need to be assessed in a systematic review to draw thorough conclusions about the validity of the evidence. Although not covered in the present paper, external validity should always be assessed in a systematic review.

### Concepts and terminology related to risk of bias

#### Internal validity (systematic error) and precision (random error) of studies

Studies that seek to estimate the true value of a quantity (such as the size of effect of an intervention or exposure on an outcome) are subject to observational error, meaning that the value of the quantity being estimated by the study will differ from the true value. Observational error has two components: systematic error (affecting internal validity; also known as bias) and random error (affecting precision) [20].

Systematic error (i.e. bias) is a consistent deviation in the results of a study from their true value, such that the study under- or over-estimates the true value. Systematic error is not determined by chance but arises from specific flaws or limitations in the design or conduct of a study.

Random error (i.e. precision), in contrast, arises from the unpredictable inaccuracy of estimation that is inherent in research studies and is reflected in results that are distributed randomly around the true value. Random error is always present in measurements to some extent. Often, random error can be reduced by increasing the sample size in a research study, or by combining the results of similar studies in a meta-analysis to increase the sample size (subject to the studies being adequately comparable), hence improving the precision of the result [21].

**Fig. 1:**
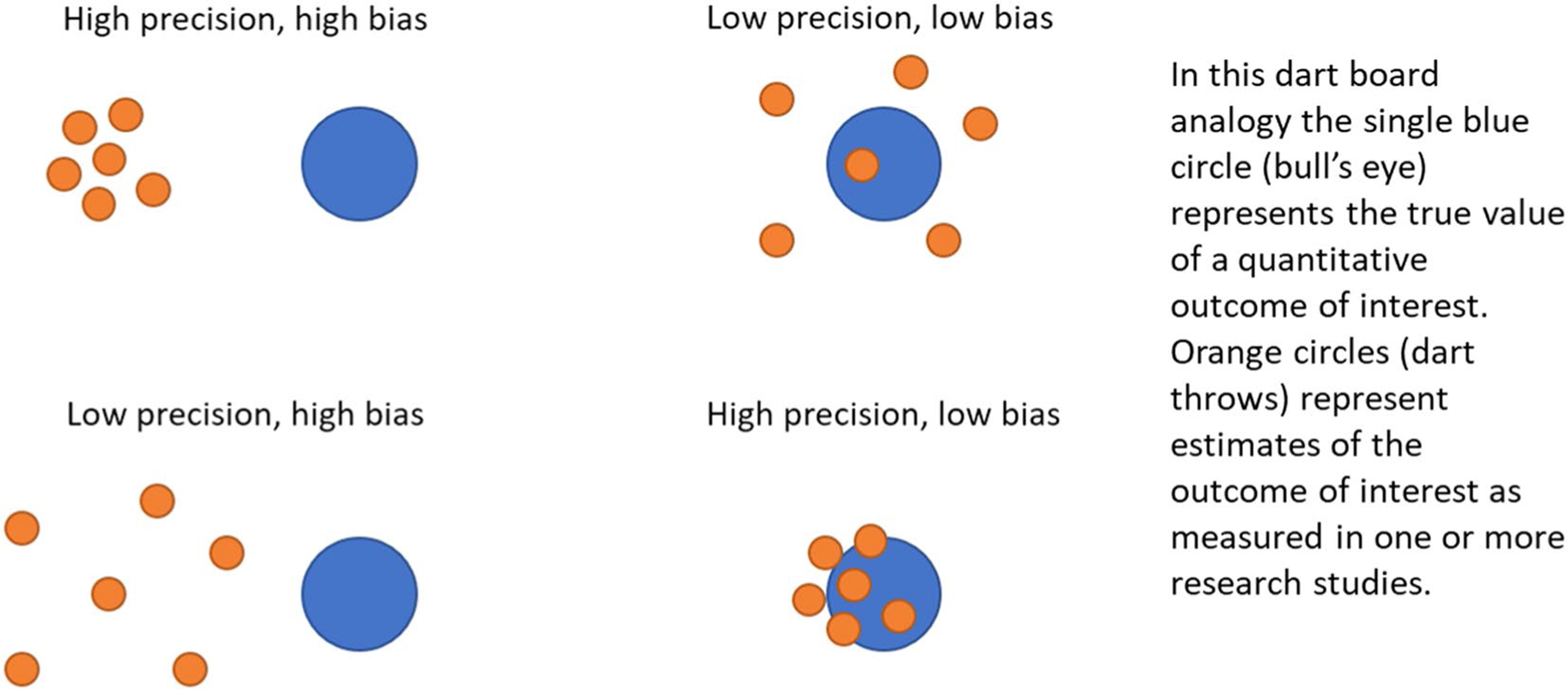
Schematic illustration of the relationship between systematic error (bias) and random error (precision)

A schematic illustration of the relationship between precision and bias is shown in Fig. 1.

Confidence intervals around outcome estimates can capture random error but not systematic error. Narrow confidence intervals may give a false sense of the validity of an outcome measure if systematic error is present but not clearly evaluated and communicated (i.e. the high precision, high bias scenario in Fig. 1).

It is important to be aware that even studies that are conducted to high standards of scientific rigor may be biased, as researchers may simply not be able to control all aspects of an experimental or observational study that introduce systematic error.

#### Internal validity and risk of bias

Internal validity refers to the degree of systematic error relating to the design and conduct of a specific study, but this cannot usually be directly measured in individual studies. Its existence has been demonstrated by large-scale research in which many studies have been collectively evaluated to investigate how different aspects of study methods influence outcomes (this research has mostly been done in medicine, where it is referred to as “meta-epidemiology” [22]). Such meta-research has shown that a range of specific deficiencies in study methods (e.g. failure to randomly sample populations or failure to account for missing observations) can introduce systematic errors in the outcomes [3, 23–30]. Whilst it is not usually possible to measure the internal validity of an individual study directly, the principles learnt from meta-research enable a judgement on whether systematic error is likely, given knowledge of the study’s methods. This judgement is referred to as the *risk of bias*.

#### Internal validity in relation to other study quality constructs

A quality construct is a distinct and evaluable aspect of the design, conduct or reporting of a study that contributes to the reliability of the study results and their interpretation, and is independent of other quality constructs. Quality constructs can be differentiated from one another by the question they address (e.g., how internally valid is a study?, how precise is a study outcome?, how well reported is a study?, or does a study meet ethical standards?).

**Fig. 2:**
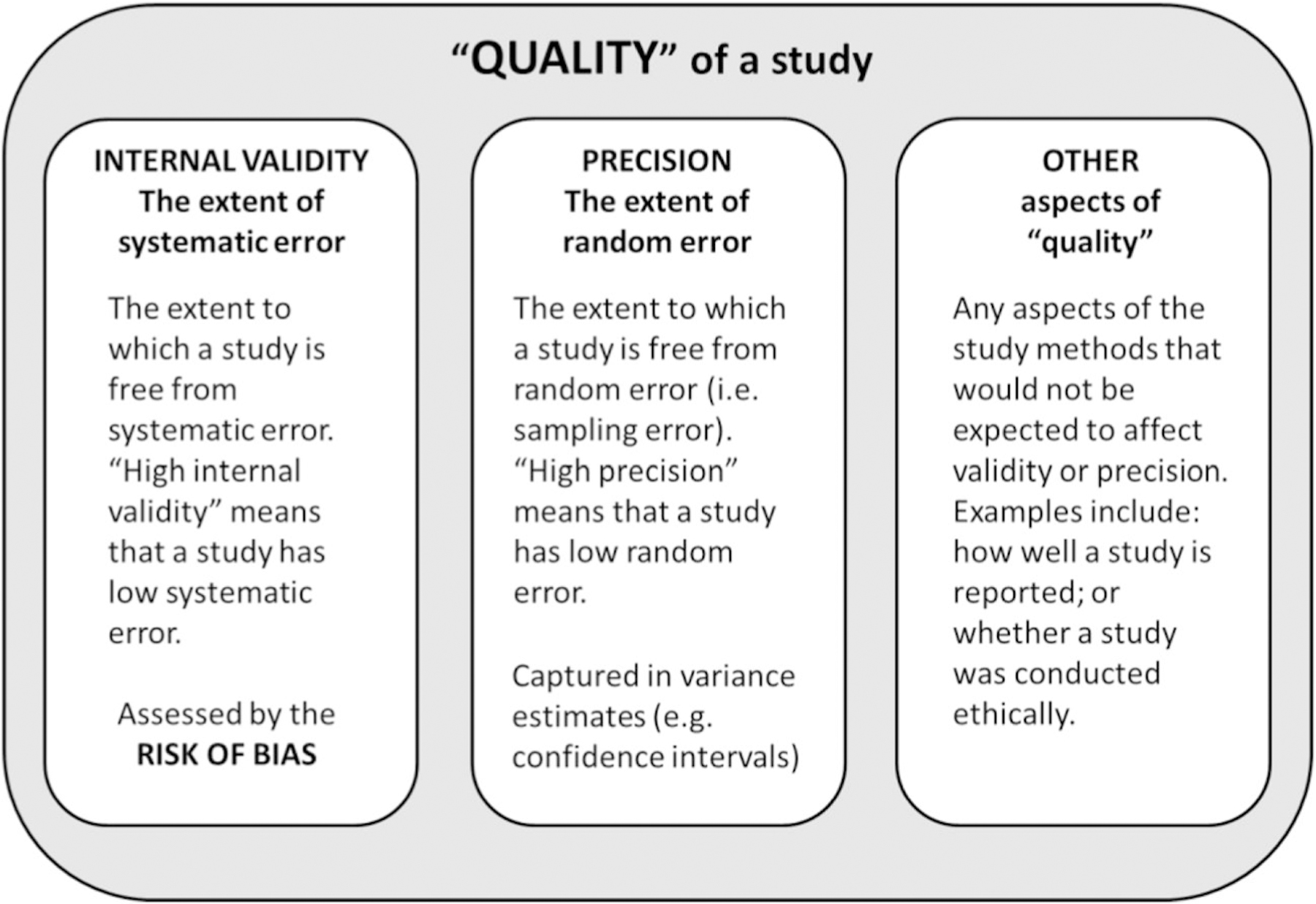
The key quality constructs of a research study

The risk of bias assessment stage of a systematic review is often referred to broadly as “quality assessment” [1], but “quality” has different meanings depending on the context [31]. The “quality” of each of the individual studies included in a systematic review may cover any or all of the following quality constructs: validity (i.e. systematic error), precision (i.e. random error), and other aspects of study design, conduct or reporting that do not relate directly to validity or precision (e.g. the extent to which a study is clearly reported or ethically sound) (Fig. 2).

When referring to study quality it is important to be clear about which quality constructs are being considered, since quality constructs differ in how they influence study outcomes and conclusions. Unfortunately, the term “quality” is often used in evidence synthesis in a broad sense without further qualification, and many critical appraisal tools are described generally as “quality assessment” tools [31–35]. We recommend that the term “quality” should not be used in evidence synthesis unless it is explicitly defined in terms of specific constructs.

### Structure of this paper

As shown in Box 1, review teams face several key challenges when assessing the risk of bias of studies included in environmental systematic reviews. The following sections of this paper aim to address these challenges as follows:

Risks of bias are explained in relation to different environmental research study designs (section “Risk of bias in relation to environmental study designs”).

The core principles of good practice for risk of bias assessment in comparative quantitative systematic reviews are introduced as FEAT: Focused, Extensive, Applied and Transparent. We explain how the FEAT principles assist in ensuring that risk of bias assessments, and any tools they are based on, are fit-for-purpose (section “Focused, Extensive, Applied, Transparent (FEAT): Core principles of critical appraisal”).

The Plan-Conduct-Apply-Report framework is presented to guide review teams on how to assess validity in quantitative systematic reviews of environmental topics, according to good practice standards in evidence synthesis (section “Risk of bias framework”).

Checklists are provided to assist with (i) planning and (ii) critiquing risk of bias assessments (section “Discussion, conclusions, limitations and checklists”).

## Risk of bias in relation to environmental study designs

Research studies can be broadly divided into experimental and observational studies. In experimental studies, the investigator has some control of the study system and conditions to reduce the effects of unintended variables on study outcomes, whereas in observational studies the investigator observes the study system and conditions without manipulating them. The extent to which studies may be susceptible to bias, and whether the risks of bias can be mitigated by using appropriate study methods, varies with the study design [3, 36]. To fully mitigate all potential sources of bias requires study designs with random acquisition of study subjects, sites or samples.

### Study designs relevant to environmental evidence reviews

There is no agreed standard inventory, lexicon, or taxonomy of the study designs employed in environmental research. Lists of study designs in environmental science and conservation [37, 38] are quite general, and do not include all study designs that could be relevant to quantitative environmental systematic reviews.

To ascertain which study designs are included in comparative quantitative environmental systematic reviews we consulted the most recently-published systematic reviews in the journal Environmental Evidence [6, 7, 10, 12, 13, 15, 39–49], as well as other relevant sources [50–54]. The study designs included in these systematic reviews are summarised in Table 1, with more detailed descriptions and examples given in Additional file 3. Some study types may be described in more than one way, for example a randomised experimental study might also be described as a before-after-control-impact (BACI) study or a control-impact study; an observational study following a specified population over time might be described as a cohort study or a longitudinal study; a survey might also be described as a cross-sectional study.

The study designs listed in Additional file 3 are not necessarily mutually exclusive, and may overlap, whilst some studies may have complex designs that include both experimental and observational elements. Randomised experiments may, for instance, include surveys or questionnaires or additional non-randomised groups. Viswanathan et al. [36] provide a flow chart for the identification and classification of observational studies; it distinguishes 13 types of study design and provides some guidance on how to classify these (e.g. how to distinguish between before-after and interrupted time series studies).

Randomised studies are not always feasible or appropriate in environmental research [55] (e.g. when large temporal or spatial scales are required to answer a question) and a wide range of non-randomised and observational study designs have been included in environmental systematic reviews (Table 1 and Additional file 3). A distinction can be made between prospective and retrospective studies. Prospective observational studies are generally less prone to some types of bias than retrospective studies, e.g. retrospective studies may be at risk of selection bias, as investigators may be able to ‘cherry-pick’ data from a set of outcomes that have already been measured (random selection could reduce this risk).

### Appraisal tools and types of bias relevant to environmental studies

#### Risk of bias tools

Many tools exist for critically appraising studies, covering a range of experimental and observational study designs [32, 34, 35, 56, 57]. Most of these tools have been developed in health research and related areas, with few available specifically for the area of environmental management and conservation [33, 37]. Not all focus on internal validity and risk of bias, even when they purport to be risk of bias tools; some are described as “quality” assessment tools. The tools often vary in the classes of bias that they cover, and some focus on randomised but not on non-randomised studies.

The process of developing risk of bias tools is constantly evolving. For instance, Cochrane introduced a new evidence-based “target study” paradigm for assessing risks of bias, in which features of the research study of interest are compared against a (hypothetical, not necessarily feasible) “perfect” randomised target study that would mitigate all risks of bias. Several risk of bias tools for non-randomised studies are now based on the target study approach, including the “ROBINS-I” tool for studies of interventions and a tool for studies of exposures [58–60]. A list of some of the commonly used risk of bias tools and checklists for a range of study designs, and the classes of risk of bias that they cover, is provided in Additional file 4 (most of these have been developed for systematic reviews on human health topics). This list is not exhaustive and will become out of date as new tools are developed. Review teams should therefore check for the latest available tools when preparing their review protocol, e.g. by conducting an Internet search and consulting relevant systematic review support resources (e.g. Systematic Review Toolbox [61]).

CEE is currently developing a Critical Appraisal Tool to aid assessment of the risks of bias in comparative quantitative environmental systematic reviews [16]. The classes of bias covered in this tool (Table 2) are based upon those identified in risk of bias tools for human health studies which are grounded in mathematical theory [58, 62]. The CEE tool is likely to be revised as new empirical evidence on sources of bias in environmental research studies becomes available [16]. We use the CEE tool [16] to illustrate the classes of bias that review teams should consider when conducting a comparative environmental systematic review addressing a PECO or PICO-type question (see “Types of bias” below). Whilst the CEE tool may be suitable for use in some systematic reviews, review teams should carefully weigh the pros and cons of available tools relevant to their systematic review (e.g. see Additional file 4) and provide a clear rationale in the review protocol for their choice of risk of bias tool(s). We provide guidance below on selecting risk of bias tools (see “Identify and use an existing risk of bias tool”), modifying existing tools if appropriate (see “Modify a near-fit tool”) and developing new tools if necessary (see “Develop a new risk of bias tool” ).

#### Types of bias

The classes of bias that should be assessed for comparative research studies, according to the most recent Cochrane tools (ROB2 [62] and ROBINS-I [58]) and the CEE tool [16] are summarised in Table 2. Further explanation of how these classes of bias may be identified in environmental research studies is provided in Additional file 5.

The term “selection bias” as widely used in the literature usually refers specifically to baseline confounding (selection before treatments). However, bias in selection can also arise later in the progress of a study and so the more recent ROB2 [62], ROBINS-I [58] and CEE [16] tools differentiate between baseline confounding and other sources of selection bias since these are mathematically distinct [59].

Ideally the naming of biases should be as intuitive and concise as possible so that their meaning can be clearly deduced and interpreted consistently. As the CEE tool [16] is currently under development, and the nomenclature may be subject to revision, we have provided the explicit naming of bias classes according to the ROB2 [62] and ROBINS-I [58] tools in Table 2, as well as the current names of the bias classes used in the prototype version of the CEE tool. Note that bias due to missing data is frequently not assessed in CEE systematic reviews (Additional file 2); we have highlighted this as a separate class of bias in Table 2 for emphasis, although strictly bias due to missing data is a type of selection bias.

It should not be assumed, as is often claimed, that observational studies by their nature will always have a higher risk of bias than experimental studies. The risks of bias for a given study are contingent on how well the study is conducted, and a poorly-conducted randomized study may not necessarily be at less risk of bias than an observational study [55].

Note that Table 2 does not include publication bias or bias arising from research sponsorship. Publication bias (preferential publication of more favourable or positive studies) applies to the overall evidence base (i.e. all studies considered together) rather than being a feature of an individual study. As such, publication bias is usually assessed separately from the risk of bias (critical appraisal) stage of a systematic review. Bias arising from research sponsorship (i.e. conflict of interest) may be important [70] but is not in itself an independent class of bias, since it would be expected that any consequences of a conflict of interest would be reflected in the classes of bias already being assessed (such as selective reporting) [55].

A wide range of types of bias have been catalogued and classified (e.g. see www.catalogofbias.org). Many of these belong to one or more of the eight classes of bias listed in Table 2 (e.g. recall biases can be categorised as detection bias, misclassification bias and/or measurement bias depending on the study design [71, 72]). The extent to which each type of bias will need to be assessed must be carefully considered when developing the systematic review protocol. Given the diversity of study designs that could be relevant, it is not possible to provide an exhaustive list of all possible biases that could occur. As explained in detail in the framework below (“Risk of bias framework” section), a logical approach for identifying relevant risks of bias is to start by determining the study design(s) that will be eligible for inclusion in the systematic review; then check whether any existing risk of bias instruments are available specifically for the study design(s) of interest and whether these are fit-for-purpose. Criteria to assist in identifying aspects of studies that would increase or decrease internal validity (i.e. increase or decrease the risk of bias) are provided in Additional file 5.

## Focused, Extensive, Applied, Transparent (FEAT): core principles of critical appraisal

### The general FEAT Principles

The core principles of critical appraisal presented here follow logically from the structure and objectives of systematic reviews and therefore all well-conducted critical appraisal assessments in systematic reviews should already adhere to them. The FEAT principles reflect overarching elements of evidence synthesis that complement and are consistent with the principles for good evidence synthesis for policy (inclusivity, rigor, accessibility and transparency) [73]. The label “FEAT” is intended to emphasise and serve as a useful reminder of the importance of these core principles. The general interpretation of these principles and their specific interpretation in relation to the assessment of internal validity is shown in Table 3.

### Applying the FEAT Principles in comparative quantitative systematic reviews

#### Focused

##### Critical appraisal should be focused on the risk of bias.

The Focused principle requires that all research projects appraising evidence should focus on quality constructs that are appropriate to the question being asked. As the aim of a quantitative systematic review is to provide an accurate estimate of one or more specified outcomes, it is imperative that the studies included in the review are checked for potential systematic errors in relation to these outcomes. It follows that quantitative environmental systematic reviews should always include an assessment of the risk of bias of the studies.

Depending on the review question, other quality constructs may also be of interest, such as how well the studies comply with a reporting guideline or whether appropriate ethical standards were adhered to. Each quality construct should be assessed separately to ensure that the risk of bias (i.e. systematic error) is not conflated with any other quality constructs.

Appropriately focusing critical appraisal is a challenge for many systematic reviews. Whilst many CEE systematic reviews have expressed the focus of their critical appraisal as being on validity or risk of bias, several reviews have conflated multiple aspects of reporting, precision, and/or validity. For example, 7 of the 10 most recently published CEE systematic reviews do not fully comply with the Focused principle (Additional file 2). By combining multiple quality constructs in this way, these systematic reviews may not have accurately characterised the potential for systematic error in the studies they included.

When focusing on risk of bias, review teams should be adequately familiar with all sources of bias that could be applicable to a study and capture these in the overall risk of bias assessment. However, a challenge for assessing the risk of bias is that, unless relevant and fit-for-purpose tools are available for the study designs and topics of interest, it may be difficult to know what specific sources of bias need to be captured. We note that each of the 10 most recently published CEE systematic reviews developed their own method of critical appraisal, presumably due at least in part to a lack of existing instruments relevant to the study designs of interest. These reviews varied considerably in the rationale they provided for the set of validity or quality criteria that they assessed (Additional file 2).

#### Extensive

##### All risks of bias should be captured in the assessment.

Quality constructs are usually complex. For example, an assessment of risk of bias should consider all the potential sources of bias that could have an important effect on the results of a study. An assessment of compliance with reporting standards should consider the different research stages of a study (methods, results, conclusions); an assessment of compliance with ethical standards should consider several component issues (e.g. whether the risk of harm is minimised, informed consent secured, and information about participants used and stored appropriately). The Extensive principle requires that all important elements of the construct must be defined and evaluated.

To comply with the Extensive principle, a critical appraisal process must address each type of bias, and correctly evaluate it, for each included study. However, 8 of the 10 most recently-published CEE systematic reviews do not fully comply with the Extensive principle, since they did not consider attrition bias, i.e. the risk of bias arising from missing data (Additional file 2).

#### Applied

##### The critical appraisal stage should inform the data synthesis appropriately

The sequence of stages in a systematic review is logical and purposeful. The critical appraisal stage precedes the data synthesis stage (which may be a narrative synthesis and/or meta-analysis) so that any threats to validity identified during critical appraisal can be considered in the data synthesis and therefore inform the conclusions of the review.

The Applied principle requires that the output from critical appraisal informs the data synthesis stage of the systematic review in an appropriate way—that is, all studies, outcomes and risks of bias identified in critical appraisal should inform the synthesis without conflating quality constructs. The appraisal process should therefore produce accurate, consistent descriptions of the extent to which each element of each construct has been fulfilled, in a form that can be logically incorporated into the overall analysis, for example in sensitivity or subgroup analyses. However, this does not always happen in practice. For example, some non-CEE environmental reviews that claimed to be systematic reviews did not conduct critical appraisal at all (Additional file 1), whilst among the most recently-published CEE reviews, several did not utilise all results of critical appraisal to inform the data synthesis, or conflated risk of bias with other quality constructs (Additional file 2).

#### Transparent

##### The critical appraisal process should be clearly reported

Appraisal judgements should be made against explicit, unambiguous criteria. The reason for each judgement should be clearly justified and transparently reported. As for any scientific study, careful, thorough documentation is critical. The Transparent principle requires enough clarity in documentation of the assessment that the critical appraisal process, which in some respects is necessarily subjective, can be followed, scrutinised, and potentially repeated by a third party.

For risk of bias assessments, explanation should be provided on how all relevant risks of bias were identified; which of those risks of bias a study was deemed susceptible to; and the rationale for the judgements made in reaching that conclusion. The system employed for classifying risks of bias (e.g. “low” or “high” risk of bias) must be clearly justified and explained. If a summary conclusion on the risk of bias is made, the process for weighing the individual risks of bias that contribute to the overall summary should be logical and clearly articulated so that specific risks of bias are neither missed nor double-counted.

We judged that, strictly, none of the 10 most recently published CEE systematic reviews fully comply with the Transparent principle (Additional file 2). In most cases this was because limited or no justification of study-level judgements on internal validity was provided; in some cases, lack of clarity around which constructs were being assessed and discrepancies between different sets of information being reported in the systematic reviews were also issues.

### Assessing risk of bias tools against the FEAT principles

For a risk of bias tool to be fit-for-purpose to inform the critical appraisal stage of a systematic review it should logically conform to the FEAT principles (Table 3). An illustration of how FEAT principles may be used to assess fitness-for-purpose of three existing risk of bias tools is provided in Additional file 6.

- FOCUSED: The tool should assess the appropriate quality construct—that is, the internal validity as assessed by the risk of bias. When checking risk of bias tools, review teams should look for a justification that the tool is evidence-based and that it has construct validity, such that it measures what it claims to measure.
- EXTENSIVE: The tool should be adequately comprehensive, such that it includes all the classes of bias relevant to a given study design. For example, a tool that assesses only selection bias would not capture all the bias classes that could arise in a study, leaving some biases unaccounted for.
- APPLIED: The output from critical appraisal should be able to directly and appropriately inform the data synthesis stage of the systematic review. For example, the Cochrane risk of bias tool (original version) [74] provides a low/high/unclear classification of risk of bias, which can be directly applied to the data synthesis: studies at high or unclear risk of bias may be included or excluded from the data synthesis in sensitivity or subgroup analyses to explore how risk of bias affects the outcomes, and hence the overall review conclusions. Where the expected direction and/or magnitude of potential bias are estimable (see “Develop a new risk of bias tool” section for further discussion) these should be captured by the risk of bias tool [55].
- TRANSPARENT: The risk of bias tool should itself be transparent, with a clear rationale and instructions for use. It should also provide a transparent output, including supporting statements to justify how risk of bias judgements were reached, to minimise subjectivity and maximise consistency of interpretation.

## Risk of bias framework

**Fig. 3:**
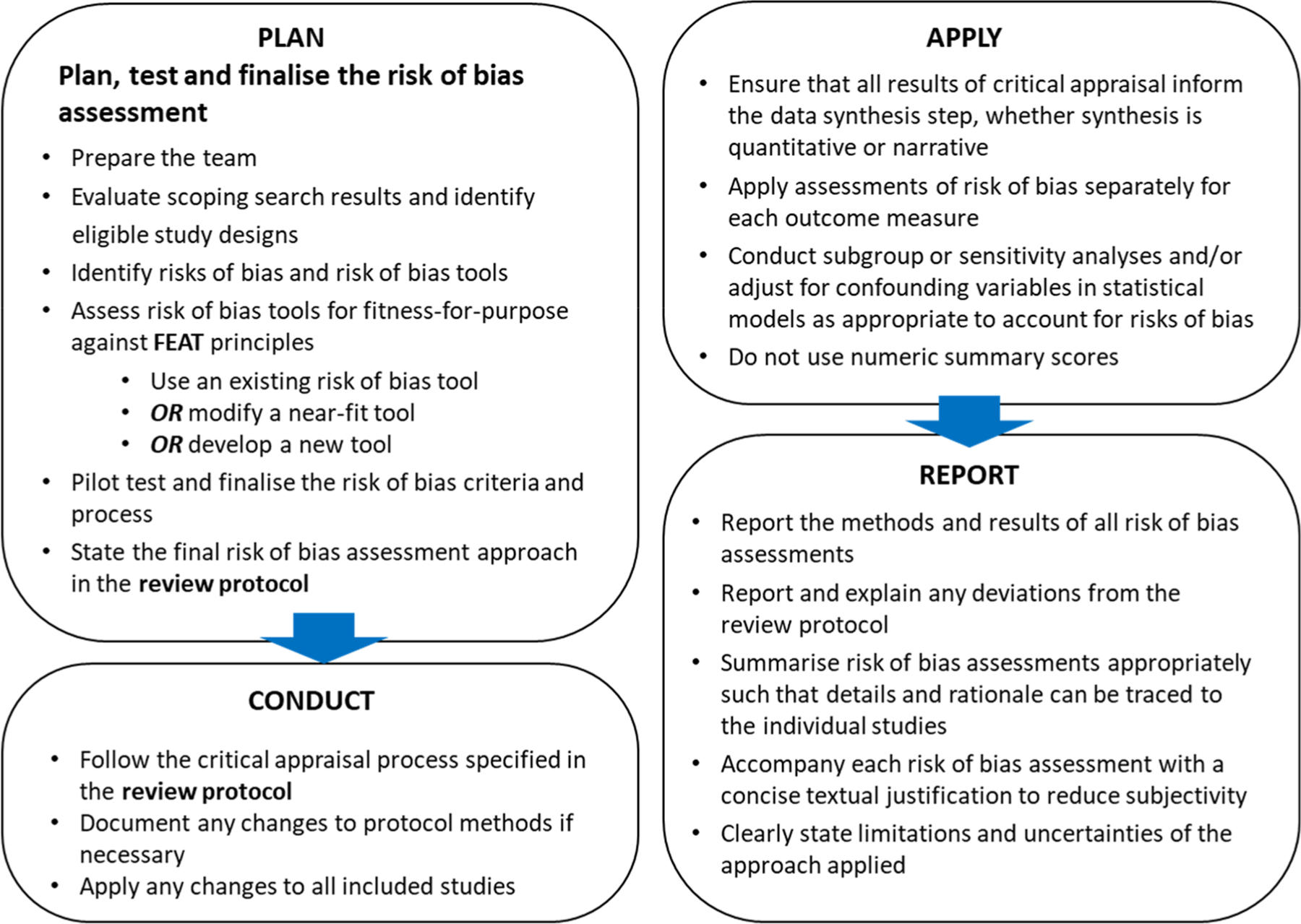
Overview of the framework for assessing risk of bias

The framework presented here aims to (1) provide consistent guidance to review teams on how to conduct risk of bias assessments in environmental systematic reviews and (2) ensure that assessments of risk of bias are performed according to the best standards of scientific practice. The framework follows a structured Plan-Conduct-Apply-Report approach (Fig. 3) and follows the core FEAT principles described above. As noted in “Scope of the paper”, the framework focuses on the internal validity (i.e. risk of bias) aspect of critical appraisal. External validity, which should also be assessed in the critical appraisal stage of a systematic review, is not covered here.

### Plan, test and finalise the risk of bias assessment

As with all stages of a systematic review, the risk of bias stage should be included in a pre-specified and peer-reviewed protocol, with further revisions made if appropriate, before the full systematic review can commence [4]. Therefore, planning the risk of bias assessment should be part of the protocol development process when planning the overall systematic review. The protocol is essential to minimise reviewer bias and make the review process as rigorous, transparent, and well-defined as possible [4].

Pilot-testing—including pilot-testing of the critical appraisal process—is especially important to ensure that the review team has adequate resources to conduct the full systematic review, and to reduce the risk of awkward methodological surprises appearing once a review has started. As with all stages of a systematic review, if any changes to the critical appraisal methodology become necessary once the full review has begun, these should be documented as protocol amendments and applied to all studies included in the review.

#### Prepare the team

A systematic review is a substantial piece of work that requires the input of a multidisciplinary team. The review team that will be conducting the critical appraisal stage should meet four key requirements: (i) The team should have enough topical expertise to be familiar with the strengths and weaknesses of research studies relevant to the research question. Review teams may benefit from having an advisory or steering group with appropriate topical and methodological expertise [4]. (ii) The team should understand the concepts of risk of bias and confounding and the ways that these are identified, assessed and reported. (iii) There should be enough team members to enable dual assessments of validity for each study. (iv) Members of the review team and advisory group should be free from potential conflicts and should not assess studies of which they are authors or contributors.

#### Evaluate scoping search results to identify relevant study designs

Scoping searches should be conducted as part of the protocol development process [4]. Scoping searches are important for determining the types of studies that are likely to meet the review’s inclusion criteria, thereby helping the review team to decide which classes of bias and risk of bias tools may be relevant. Scoping searches can also provide an indication of the volume of evidence that may need to be critically appraised, and hence the likely resource requirements for the risk of bias assessment and other stages of the review.

Studies should be labelled and described in a concise way that provides as much information as possible, as this maximises transparency and will help to inform which risk of bias tools may be applicable. The description should indicate whether the study is prospective or retrospective, the nature of any comparison being made (e.g. before-after, control-impact or both), and whether the study units (e.g. population groups or study plots) are determined selectively or randomly.

#### Identify risks of bias and risk of bias tools

##### Consult with topic experts and other stakeholders

An adequate understanding of how to answer environmental questions requires specialist knowledge, in topics such as ecology, toxicology, agronomy, taxonomy, and/or statistics. Consultation of review teams with experienced topic experts and other stakeholders may help to ensure that all key risks of bias are considered. However, only four of the 10 most recently published CEE systematic reviews reported that they had engaged with an advisory team [6, 15], held meetings with subject experts [11], or held a stakeholder workshop [13] (Additional file 2).

A clear record should be kept of how consultation with stakeholders is managed, indicating who the stakeholders are, the process used for consultation (including any arbitration required), and any changes made to the review protocol as a result of the consultation process.

##### Develop conceptual models for each outcome

Conceptual models, including logic models and directed acyclic graphs, provide a visual aid to scientific discussion by making underlying relations and causal assumptions explicit. Conceptual models have potential to support the development and refinement of the risk of bias stage of a systematic review, although we note that the most recently published CEE systematic reviews (Additional file 2) have not explicitly used conceptual models for this purpose. A conceptual model could, for example, clarify how organisms of interest interact with one another and with the exposures of interest and any co-exposures likely to be present, and indicate any direct and indirect effects that could occur [75, 76].

Directed acyclic graphs can help to identify the presence of confounding factors for a given question (Table 2), potentially assisting with the identification of a minimum set of factors to eliminate confounding [77].

During development of a conceptual model, sources of confounding that are considered by the review team and topic experts to be unimportant, and those that would be controlled for in the study design(s) that are being critically appraised, can be excluded, but should be clearly documented.

By seeking expert opinion from within the review team and topic experts it should be possible to further develop a core conceptual model if necessary, until the review team is satisfied that all key factors, processes and interactions relevant to the review question have been considered.

##### Consider the “perfect” target study design as a reference benchmark

When seeking possible sources of bias associated with a particular outcome measure it may be helpful to visualise what the “perfect” study design (“target study design”) would need to be to mitigate all the threats of bias and confounding that could arise. This approach has been adopted by recent risk of bias tools (such as the Cochrane ROBINS-I tool [59]).

There is currently no formal guidance available on how to visualise what the perfect target study design would be for a given outcome measure of interest. However, this can be approached systematically by asking how a study would need to be designed and conducted to eliminate each of the classes of bias listed in Table 2 for each outcome measure of interest. This may be an iterative process, drawing upon topic expertise of the review team, as well as expertise of stakeholders and other topic experts (experts in experimental design and statistics). It is particularly important that all possible sources of confounding are identified. The “perfect” study design need not be realistic; its purpose is simply to identify how real-world decisions and constraints in designing a study may introduce systematic error into its results.

For example, to minimise the risk of selection bias the target study design would require random selection or random allocation of study participants, sites and/or samples (or if observational in design, a perfect match between study groups might be a preferable “ideal” approach to visualise). To minimise the risk of several classes of bias the target study design should ensure that the study investigators and, if applicable, human participants cannot determine the identity of the exposure and comparison groups or samples, which may be achieved by blinding/masking (Box 2).

Once the target study design has been determined it can then serve as a reference against which to assess any shortcomings of the study design(s) of interest to the systematic review. Among the most recently published CEE systematic reviews, two have considered what would be the “ideal” or “gold standard” study that could answer their primary question in an unbiased way, assuming that resources and field conditions were unlimited [13, 15], although they reported limited details of these target studies.

The target study design may be more immediately obvious for some types of studies than others. For example, a well-conducted randomised ecological experiment may already meet most, if not all, the requirements to minimise all the classes of bias listed in Table 2. A retrospective observational monitoring study is quite different from a randomised experimental study, but it should nevertheless be possible to visualise a “matching” target study design that would minimise the risks of bias. For instance, the target study design would require acquisition of the retrospective samples to be random to minimise the risk of selection bias. Similarly, random selection of participants and/or responses is preferable to minimise selection bias in surveys. In some cases, the target study design may indicate an implausible action is necessary to prevent bias. For example, accurate recollection of events is a problem in surveys (recall bias) and the target study design would require human respondents to have perfect memory, which is very unlikely in real life. However, steps may be taken to reduce the risk of recall bias, for example, by validating recall against objective measurements of events, or requiring recall over only a short timescale.

#### Assess risk of bias tools for fitness-for-purpose against FEAT principles

##### Identify and use an existing risk of bias tool

Once the sources of bias and confounding and the study designs needed to mitigate these have been identified, the review team may use an existing risk of bias tool (if one exists that is fit-for-purpose), modify an existing tool, or develop a new tool.

If the review team intend to use an existing risk of bias tool, it should be checked to ensure that it meets the FEAT principles described above (see “Assessing risk of bias tools against the FEAT principles” above). An illustration of how a risk of bias tool can be checked for fitness-for-purpose against the FEAT principles is provided in Additional file 6.

Note that the tools listed in Additional file 4 vary in their length, complexity, and the extent of explanatory guidance provided. Some risk of bias tools may be easier and less time-consuming to use than others, and the review team should determine the resources that will be needed for conducting the risk of bias assessment when pilot-testing the process.

##### Modify a near-fit tool

Risk of bias tools may not be available for some types of study design or may not be considered fit-for-purpose if they do not meet one or more of the FEAT principles. It should not be assumed that just because a tool has been used previously, or widely, that it is appropriate for use.

If risk of bias tools do not cover the study design(s) of interest, or existing tools are not deemed sufficiently fit-for-purpose, then review teams may consider modifying one or more existing tools. To modify (i.e. improve) an existing near-fit risk of bias tool, it will first be necessary to establish which of the FEAT principles (i.e. Focused, Extensive, Applied and/or Transparent) the tool fails to include (see “Assessing risk of bias tools against the FEAT principles” above). To avoid plagiarism or infringement of copyright, review teams should clearly reference the original tool and check whether the tool has any copyright restrictions that would preclude modifying it (note that some Cochrane tools carry a CC-BY-ND licence which would prohibit creating any derivatives of the tool).

If an existing tool is deficient in the Focused and Extensive principles of FEAT (i.e. it does not appear to capture all relevant sources of bias), then the review team should extend the tool to include the missing sources of bias and ensure that internal validity is not conflated with other constructs. The updated tool should then be pilot-tested and iteratively refined as needed to ensure consistency of interpretation and use by the review team.

If a near-fit tool is deficient in aspects of the Applied or Transparent criteria, then different aspects of the existing tool may require modification. For instance, if the tool appears to identify all relevant sources of bias and confounding (based on a logical and plausible rationale, or on evidence from large-scale research) but the tool output is unsuitable for informing the data synthesis (e.g. if the output conflates bias with other aspects of study quality, or if it combines multiple judgements for different classes of bias into a single score), then framing the output format will require updating and pilot-testing.

When an existing near-fit risk of bias tool is modified by the review team, a clear explanation of the rationale should be provided in the review protocol. This explanation should indicate how the modified tool differs from the original version and how it was determined to be fit for purpose after modification and pilot-testing.

##### Develop a new risk of bias tool

Before attempting to develop a new risk of bias tool, the review team should first check whether any fit-for-purpose risk of bias tools exist for the study design(s) of interest (“Identify and use an existing risk of bias tool” section) and whether any tools that are not fully fit-for-purpose are suitable for modification.

###### (i) Specify the relevant classes of bias

As suggested above, a logical starting point for a review team planning a risk of bias assessment is to consider whether the study designs of interest would be susceptible to the classes of bias listed in Table 2, plus any other sources of bias that may be relevant to the study design, as identified through careful consideration including stakeholder engagement (see “Consult with topic experts and other stakeholders” above) and conceptual models (see “Develop conceptual models for each outcome” above). The classes of bias finally selected would form the basis of a new risk of bias tool. If more than one study design will be included in the systematic review, then the review team will need to consider whether to develop a separate risk of bias tool for each study design, or a tool capable of accommodating more than one study design.

###### (ii) Specify the format for classifying, recording and reporting the risk of bias

Having identified the classes of bias that a risk of bias tool should cover, the tool will need to provide a means of reporting these risks of bias in a format that can directly inform the data synthesis. Importantly, the output of the tool should not conflate internal validity with other quality constructs. Examples of approaches for classifying the risk of bias in some existing risk of bias tools are shown in Table 4.

As a starting point, review teams could consider whether a simple (e.g. high/low/unclear) risk of bias categorisation would be appropriate for their review. As an alternative, review teams could consider a more detailed classification (e.g. definitely high/probably high/definitely low/probably low/no information) if this helps to reduce the number of judgements that are “unclear.” Several possible approaches are available, each with different advantages and limitations (Table 4).

When planning the risk of bias assessment classification, it is important to consider how the categories will be summarised across the bias classes such that a risk of bias judgement for the study as a whole can inform the data synthesis (see point (v) below). In general, a classification with few categories will be more straightforward to summarise. The risk of bias categories and the process for summarising them across bias classes should make logical sense and should not be arbitrary.

Numerical scores are sometimes employed for summarising risk of bias categories. However, categorical judgements with explanations provide better information about bias for a given study. Numeric scores are inadvisable for summarising risk of bias for several reasons (Box 3).

###### (iii) Estimating the direction and magnitude of potential bias

A limitation of most risk of bias tools is that they do not capture the likely direction and magnitude of any bias, but merely summarise the risk of bias categorically [55]. It is often not straightforward to estimate the direction and magnitude of potential bias. However, where possible review teams should attempt to identify the expected direction of each class of bias (i.e. whether outcome estimates would be under-or over-estimated or effect estimates driven towards or away from the null) and estimate the likely relative magnitude of such an influence. A relative estimate of the magnitude of bias compared to the anticipated magnitude of the treatment effect may indicate that the risk of a given class of bias can be regarded as negligible [55]. Information on the likely direction and magnitude of potential bias would improve a systematic review by enabling more detailed sensitivity analyses to explore the impact of potentially biased studies on the review conclusions. Steenland et al. 2020 [55] discuss approaches for estimating bias direction and magnitude, with examples. However, if a review team is unable to estimate the direction or magnitude of a potential bias, they should not make an uninformed guess.

###### (iv) Specify signalling questions

Signalling questions are questions in a checklist (or flow diagram) for a risk of bias tool which ask about aspects of study design that mitigate or increase the risk of bias, to reach a risk of bias judgement. The questions should be phrased such that they clearly distinguish between each category of bias risk (such as those shown in Table 4) for each class of bias covered. Examples of signalling questions and their supporting instructions are available in the Cochrane risk of bias tools [58, 59, 74, 81] and the CEE tool [16]. Signalling questions and any accompanying instructions should be pilot-tested and revised if necessary when developing the systematic review protocol to ensure that the review team can be consistent in applying the risk of bias classification across studies.

As well as asking signalling questions, a risk of bias tool should ask the assessor to provide a concise statement explaining the rationale for each risk of bias judgement. This is important because risk of bias judgements inevitably involve subjectivity.

###### (v) Specify the process for summarising the overall risk of bias

Careful thought should be given to how the risk of bias will be summarised across the classes of bias in a study to give a meaningful overall risk of bias classification for the outcome of interest that can inform the data synthesis. Some risk of bias tools include guidance and provide algorithms which combine the answers to signalling questions for each class of bias to suggest a conclusion on the risk of bias for the outcome of interest [16, 59, 81]. Examples of study-level conclusions on risk of bias are shown in Table 5. If information on the expected direction and/or magnitude of bias is available for one or more classes of bias this should be considered when determining the overall risk of bias for the outcome [55].

#### Pilot-test and finalise the risk of bias assessment criteria and process

Pilot-testing—an iterative process in which the draft protocol is gradually improved until it is suitable for supporting a systematic review without further modification—is an essential part of the development of a review protocol. Pilot-testing:

- Provides an indication of flow long the validity assessment will take, thereby assisting with planning the full systematic review.
- Enables agreement between reviewers to be checked; if agreement is poor this should lead to a revision of the instructions or signalling questions.
- Provides training for the review team in how to interpret signalling questions and apply the classification criteria.
- Enables unanticipated issues to be identified and dealt with before the methods are finalised.

#### State the final risk of bias assessment approach in the review protocol

Systematic reviews should always be performed according to an a priori protocol, and amendments to the protocol should be avoided if possible. This is to ensure that methods are followed objectively, reducing the risk that “drift” of methods could introduce bias in the review process. However, if an amendment to the critical appraisal criteria or process is necessary, the revised method should be applied to all studies included in the systematic review and a transparent explanation of any deviations from the protocol should be provided in the final review report.

Guidance on how to report a systematic review protocol is available in the scientific literature, for example CEE recommend using the ROSES checklist [82]; however, this lacks specific detail of how to report the risk of bias assessment. We have therefore listed the key items that should be stated in the critical appraisal section of the review protocol, and the rationale for each item, in Table 6.

### Conduct

The systematic review should be performed according to methods specified in the protocol [4]. Protocol-specified methods aim to ensure that all included studies are treated fairly and consistently, following agreed best-practice methods to reduce the risk of introducing bias and unnecessary subjectivity.

If any deviations from the protocol are required, all changes in the methodology should be applied to all included studies of the relevant design, to ensure that the studies are treated fairly and consistently and to minimise the risk of introducing bias [4]. This will require reassessment of studies that had previously been assessed before the amendment was made.

### Apply

The output of the risk of bias assessment should be a logical and intuitive classification of the studies for each outcome that will directly inform the data synthesis. If a quantitative synthesis (i.e. meta-analysis) is possible, sensitivity analyses and/or subgroup analyses should be conducted to investigate whether and how the review’s conclusions would vary according to the risks of bias of the included studies. Statistical methods may be employed to adjust for some types of bias in the meta-analysis (e.g. using Bayesian modelling techniques [83]). If the review team intend to do this, then the approach employed should follow methods that have been planned, pilot-tested, and specified in the review protocol.

If only a narrative synthesis is possible (e.g. due to heterogeneity of the studies), then the review team should ensure that the narrative synthesis is clearly structured, so it is obvious how studies with different risks of bias contribute to the review’s conclusions (e.g. by structuring the analysis according to risk of bias subgroups).

Specific recommendations for applying risk of bias assessments to inform the data synthesis are shown, with an explanation of the rationale, in Table 7.

As many studies as possible should be retained for the data synthesis. Methods such as sensitivity analysis and triangulation are preferable for considering the overall effects of possible biases, rather than excluding high risk of bias studies by default [55].

### Report

Having assessed the risk of bias and confounding and applied the results to inform the data synthesis, the methods and results of the critical appraisal exercise, together with any limitations encountered, should be reported in the final systematic review report. Detailed methods will have been specified in the review protocol (see Table 6) and the final systematic review report may cite the review protocol for these methods rather than repeating them. However, if any deviations from the protocol-specified methods were necessary these should be clearly stated in the final review report, together with an explanation and confirmation that any changes to the protocol were applied to all included studies of the relevant design in the review.

Specific recommendations for reporting the risk of bias assessment are provided in Table 8.

## Discussion, conclusions, limitations and checklists

### Discussion and conclusions

The present paper provides a structured and systematic approach to address an unmet need for guidance to review teams on planning, conducting and reporting risk of bias assessments in comparative quantitative environmental systematic reviews. The FEAT principles and Plan-Conduct-Apply-Report framework reflect overarching principles of good practice in evidence synthesis that are independent of the topic area. The present paper thus may be of interest to review teams working across a range of topics and is not necessarily limited to systematic reviews on environmental questions.

Although CEE systematic reviews represent a high standard of evidence synthesis in the areas of environmental management and conservation, there is a need for improvement in the planning, conduct and reporting of critical appraisal in CEE reviews (Additional file 2). This need is even stronger among non-CEE systematic reviews, most of which, according to our random sample, do not have a critical appraisal stage (Additional file 1). Raising awareness of the rationale and need for robust critical appraisal in systematic reviews will be paramount for improving the overall standards and fitness-for-purpose of systematic reviews in environmental research. We encourage scientific journals that publish systematic reviews to consider whether the summary checklists we provide for planning and critiquing risk of bias assessments (see “Checklists” section below) could support editors, peer reviewers and authors to ensure that published systematic reviews meet adequate standards to inform decision making. For example, the FEAT mnemonic is referenced in the CREST_Triage tool (https://crest-tools.site/) used by the journal Environment International for editorial screening of systematic review manuscripts.

#### Potential of the FEAT principles to improve evidence synthesis

The FEAT principles presented in this paper can be used to: assist the planning and conduct of risk of bias assessments in systematic reviews; assess the completeness of existing risk of bias assessments; and assess the fitness-for-purpose of risk of bias tools. The FEAT principles could also have utility in facilitating discussions of which (if any) elements of risk of bias assessment should be included in rapid evidence syntheses (e.g. by providing a structured framework for stakeholders to consider which classes of bias and confounding can justifiably be omitted in a rapid review). We note that currently many rapid reviews appear to omit any consideration of the risk of bias [85, 86], despite the possibility for bias to influence the review’s conclusions [87]. Clearly a balance needs to be struck between what should be assessed in a rapid review and what is feasible given resource constraints and the sensitivity of the rapid review question to bias [80]. However, as an example, the current DEFRA and NERC guide for rapid evidence assessments of environmental topics [88] appears not to meet any of the FEAT criteria, as it conflates different quality constructs, lacks transparency in definitions, and does not provide guidance on how or whether any assessment of validity should inform the conclusions [89].

#### Blinding in environmental studies

Blinding has rarely been performed in environmental studies [26, 78] and no systematic review published in the journal Environmental Evidence has discussed whether blinding/masking of study investigators or participants in the included studies would have been necessary (under ideal circumstances) to mitigate against bias. This likely reflects that blinding/masking has not been considered in any of the studies included in the environmental systematic reviews. Blinding/masking is routinely assessed in risk of bias assessments for human health research and researchers should also consider the need for and feasibility of blinding/masking in environmental studies (Box 2).

#### Automation of risk of bias assessments

The critical appraisal stage of a systematic review can be resource-intensive [90, 91]. Tools are therefore being developed to automate at least parts of the process, for example by using machine learning approaches [92, 93]. In theory, when such tools become adequately reliable, an automated risk of bias assessment could replace one or more of the human reviewers who conduct a risk of bias assessment. At present the available tools (e.g. RobotReviewer [92]) are limited to specific study designs such as randomised controlled trials and do not extract all the classes of bias and confounding listed in Table 2. If automated tools are to be employed to support critical appraisal, then it is essential that review teams adequately pilot-test these tools to ensure that they can correctly identify, classify and report different risks of bias in a manner consistent with the FEAT principles.

#### Recommendations for research

During the preparation of this paper several areas were identified where further research would be helpful:

- Investigation of why critical appraisal is often not conducted in environmental systematic reviews. For instance, is this due to lack of awareness of the need for critical appraisal; the mislabelling of reviews as being systematic when they are not [94]; or poor enforcement of basic standards of rigor during the review publication process (involving editors, authors and/or peer reviewers)? Appropriate solutions will depend on the causes.
- Development of a lexicon or classification of the study designs relevant to environmental systematic reviews, to assist review teams in being consistent when discussing and reporting these, and to support the development of risk of bias tools.
- Guidance to support review teams on how to estimate the likely direction and magnitude of potential bias [55], with examples for environmental studies.
- Guidance to support review teams on methods for correcting for bias, with examples for environmental studies.

### Limitations of this paper

External validity (i.e. the relevance of a study to the systematic review question) should always be assessed in a systematic review. However, assessment of external validity is outside the scope of this paper. In principle, the overarching Plan-Conduct-Apply-Report process presented for critical appraisal would be applicable for any type of validity assessment. The main difference between assessments of internal validity and external validity lies in the range of sources of systematic error that can arise and how these are identified. Arguably this is less complex to assess for external validity since the PICO/PECO elements of the studies and of the review question provide a rational and relatively straightforward structure for considering where systematic differences may arise [19, 95].

The scope of this paper also does not include publication bias. As noted above (“Risk of bias tools” section), this is because publication bias is assessed for the overall body of evidence, not at the level of individual studies. Where possible, publication bias should always be assessed in a systematic review.

In summary, whilst risk of bias assessment is key part of the assessment of the validity of studies and is the focus of this paper, external validity, publication bias and other factors such as biological plausibility [55] also need to be considered when formulating the conclusions of a systematic review.

### Checklists

To assist review teams when planning, conducting and critiquing risk of bias assessments the following checklists are provided, based on the FEAT principles:

- Checklist for planning a risk of bias assessment—Additional file 7.
- Checklist for critiquing a risk of bias assessment—Additional file 8.

## Supplementary Material

s1 **Additional file 1.** Frequency of critical appraisal in a random sample of 50 environmental management systematic reviews.

s2 **Additional file 2.** Overview of critical appraisal approaches employed in the ten most recent CEE systematic reviews up to July 2021.

s3 **Additional file 3.** Examples of study designs that have been included in environmental systematic reviews (SR) and meta-analyses (MA) addressing PECO or PICO-type questions.

s4 **Additional file 4.** Examples of risk of bias tools and checklists for specific study designs.

s5 **Additional file 5.** General criteria for considering risks of bias in comparative quantitative environmental research studies.

s6 **Additional file 6.** Examples of critical appraisal instruments assessed for fitness for purpose against the FEAT principles.

s7 **Additional file 7.** Checklist for planning a risk of bias assessment.

s8 **Additional file 8.** Checklist for critiquing a risk of bias assessment.

## Figures and Tables

**Table 1:** Overview of the broad types of study included in comparative environmental systematic reviews addressing PECO/PICO-type questions (for detail see Additional file 3)

Experimental studies	Randomised controlled experiments Non-randomized controlled experiments	e.g. before-after-control-impact (BACI) studies, control-impact studies, before-after (BA) studies
Observational studies	Case–control studies Temporal monitoring studies Cross-sectional studies	e.g. non-intervention spatial comparison studies e.g. prospective or retrospective time series studies e.g. surveys, questionnaires, interviews; studies of spatial occurrence, distribution or prevalence (may be embedded within other study designs)
Other study types	Combined experimental and observational studies	Wide range of designs possible

**Table 2:** Classes of bias and their general interpretation

Risk of bias class	Summary (for further details and examples see [16] and Additional file 5)
1. Bias due to confounding (prior to occurrence of the exposure)	Referred to as “risk of confounding biases” in the CEE tool [16]. These biases arise due to one or more uncontrolled (or inappropriately controlled) variables (confounders) that influence both the exposure and the outcome. If there is confounding then the association between the exposure and outcome will be distorted Potential confounders may be identified by exploring whether characteristics of the study population (e.g. morphological or physiological differences between individuals, such as colour, age or sex; or characteristics of study plots) are predictive of the outcome effect of interest. Causal directed acyclic graphs (DAG; also known as causal models or causal diagrams) can be a useful tool for investigating the potential of confounding [63, 64]. Randomisation may be used to control confounding but it should not be assumed that randomisation was successfully implemented (e.g. baseline differences between characteristics of the exposure and comparator groups could be suggestive of a problem with the randomisation process [59])
2. Bias in selection of subjects/areas into the study (at or after initiation of the exposure and comparator) (commonly referred to as selection bias)	Referred to as “risk of post-intervention/exposure selection biases” in the CEE tool [16]. These biases arise when some eligible subjects or areas are excluded in a way that leads to a spurious association between the exposure and outcome, that is, selection bias occurs when selection of subjects or study areas is related to both the exposure and the outcome [62]. Selection bias can arise by unconscious or intentional selection of samples or data such that they confirm or support prior beliefs or values of the investigator (also called confirmation bias). Systematic differences in the selection of subjects or areas into the study can also be caused by missing data, if there is differential missingness between the study groups, and therefore bias due to missing data is a type of selection bias. The CEE tool includes bias due to missing data as a post-intervention/exposure selection bias [16]. We have highlighted bias due to missing data separately, consistent with the ROB2 [62] and ROBINS-I [58] tools, for reasons explained below under bias class 5 “bias due to missing data”
3. Bias due to misclassification of the exposure (observational studies only—see class 4 below for experimental studies)	Referred to as “risk of misclassified comparison biases” in the CEE tool [16]. These bases arise from misclassification or mismeasurement of the exposure and/or comparator which leads to a misrepresentation of the association between the exposure and the outcome (also known as measurement bias or information bias [65]). Accurate and precise definitions of exposure and comparator groups are necessary for avoiding misclassification
4. Bias due to deviation from the planned exposure (intervention) in experimental studies (also called performance bias) (experimental studies only—see class 3 above for observational studies)	Referred to as “risk of performance biases” in the CEE tool [16]. These biases arise from alteration of the planned exposure or comparator treatment procedure(s) of interest after the start of the exposure, when the subjects or areas of interest continue to be analysed according to their intended exposure treatment Deviations from the planned exposure could include the presence of co-exposures/co-interventions other than those intended; failure to implement some or all of the exposure components as intended; lack of adherence of subjects or areas to the intended exposure protocol; inadvertent application of one of the studied exposure protocols to subjects or areas intended to receive the other (contamination); and switches of subjects or areas from the intended exposure to other interventions/exposures (or to none)
5. Bias due to missing data (also called attrition bias)	Bias due to missing data can be considered as a type of selection bias; in the CEE tool [16], bias due to missing data is included in the “risk of post-intervention/exposure selection biases” (i.e. bias class 2 above). We have highlighted bias due to missing data separately here to raise awareness of the importance of checking studies for missing data, given that 8 of the 10 recently-published CEE systematic reviews did not consider risks of bias due to missing data (Additional file 2) Risks of bias due to missing data can arise when later follow up data of subjects or areas that are initially included and followed in the study are not fully available for inclusion in the analysis of the effect estimate. The risk of bias depends on there being (i) an imbalance in the amount of missing data between the exposure and comparator groups (differential missingness); (ii) the reason(s) for the data being missing being related to the exposure or the outcome; and (iii) the proportion of the intended analysis population that is missing being considered sufficient that the bias would substantively influence the effect estimate [58, 65]
6. Bias in measurement of outcomes (also called detection bias)	Referred to as “risk of detection biases” in the CEE tool [16]. These are biases arising from systematic differences in measurements of outcomes (also known as measurement bias [65]). Systematic errors in measurement of outcomes may occur if outcome data are determined differently between the exposure and comparator groups, either intentionally (e.g. influence of desire to obtain a certain direction of effect) or unintentionally (e.g. due to cognitive bias or human errors). When studying complex systems, and especially when many steps are involved in measuring outcomes, each calibration method or applied instrument may need to be the same between groups; if any devices or their measurements differ between study groups this may introduce bias [66]
7. Bias in selection of the reported result (also called reporting biases)	Referred to as “risk of outcome reporting biases” in the CEE tool [16]. These are biases arising from selective reporting of study findings. Selective reporting may appear at three different levels [62]: (i) presentation of selected findings from multiple measurements; (ii) presentation of results for selected subgroups or subpopulations of the planned analysis population; and (iii) presentation of selective findings from multiple analyses
8. Bias due to an inappropriate statistical analysis approach (may also be called statistical conclusion validity)	Referred to as “risk of outcome assessment biases” in the CEE tool [16]. These are biases due to errors in statistical methods applied within the individual studies included in a systematic review. There is currently no such bias class in widely applied risk-of-bias assessment tools in medicine and health research (RoB 2 [62] and ROBINS-I [58]) although it has been argued that this is an important source of bias that should be considered [55]. Issues with statistical validity can be divided into four main areas: (i) data analysts’ awareness of the exposure or comparator received by study subjects or areas (blinding of data analysts could mitigate the risk of bias); (ii) errors in applied descriptive statistics (e.g. miscalculation of sample sizes, means, or variances, including pseudoreplication [67]); (iii) errors in applied inferential statistics (including flawed null hypothesis testing, estimation, or coding); (iv) use of inappropriate statistical tests or violation of assumptions required by tests (e.g. criteria for normality and equal variances are not satisfied)
9. Other risks of bias	Any risks of bias or confounding pertinent to the study design(s) of interest that are not covered in the eight classes of bias above. Includes risks of bias that are inherent to specific study designs such as test accuracy studies [68, 69]

**Table 3:** The general FEAT principles for critical appraisal of studies in evidence reviews and their specific interpretation for assessing threats to internal validity (risk of bias)

Principle	General interpretation for critical appraisal	Interpretation in relation to assessing threats to internal validity (risk of bias)
FOCUSED	Critical appraisal should be directed at key quality constructs that are relevant to the evidence review. Each construct should be appraised separately	In comparative quantitative systematic reviews, internal validity should always be assessed; the assessment of internal validity should be separate, not conflated with other quality constructs
EXTENSIVE	All important elements of the target quality construct should be identified and evaluated	All relevant threats to internal validity (i.e. all important individual sources of bias and confounding) relevant to the studies being assessed should be included in the appraisal
APPLIED	The appraisal process should logically inform the data synthesis, with accurate, consistent descriptions of the extent to which each element of each construct has been fulfilled	The internal validity assessment should inform the data synthesis in an appropriate format (e.g. to support sensitivity or subgroup analyses)
TRANSPARENT	Judgements should be made against explicit, unambiguous criteria. The reason for each quality judgement made by the reviewers should be clearly justified and transparently reported	All internal validity judgements should be based on pre-specified and agreed criteria detailed in the review protocol; each judgement should be supported with a concise explanation and grounded in evidence of the practices used in the study that is being appraised

**Table 4:** Possible ways of classifying risks of bias to inform data synthesis

Risk of bias classification	Advantages and limitations
Low risk/ high risk/unclear risk	This approach has been used extensively in the original Cochrane risk of bias tool for randomised controlled trials [74, 79] and has the advantage that the results are easy to tabulate or present graphically (e.g. using a “traffic light approach” in which red = high, green = low; amber = unclear). However, a disadvantage of having an “unclear” category is that it may be tempting for reviewers to be less decisive and assign most studies to this category
Definitely low risk/probably low risk/probably high risk/definitely high risk	This approach has been proposed by OHAT [80] as a way to avoid having an “unclear” or “no information” category by requiring that instances of insufficient information are recorded within the “probably high risk” category”. Note that OHAT provides explicit criteria defining each category in their guidance [80]
Low risk/moderate risk/serious risk/critical risk/no information	This approach has been used in the latest version of the Cochrane risk of bias tool for randomised controlled trials, with explicit definitions of each category [81]. The categories of “low” and “moderate” risk of bias in Cochrane’s classification are intended to be specifically interpreted in relation to how well the study in question matches an ideal target study design. We also note that several published environmental management systematic reviews included a “medium” risk category (Additional file 2). Where authors use categories such as “medium”, “moderate”, “critical” or “serious”, these should be clearly defined to minimise discrepancies in interpretation

**Table 5:** Examples of study-level risk of bias classifications

Risk of bias classification for each class of bias	Possible study-level risk of bias classification for a given outcome of interest
All classes of bias are judged to have low risk of bias, definitely low risk of bias, or probably low risk of bias for the outcome of interest	Low risk
At least one class of bias is judged to have high risk of bias, definitely high risk of bias, or probably high risk of bias for the outcome of interest	High risk
Some classes of bias are judged to have low risk, others unclear risk, but no classes are judged to have high risk for the outcome of interest	Unclear risk (to avoid an unclear judgement for the overall study it is preferable for each domain to reach a probably low risk or probably high risk judgement instead of an “unclear” judgement, where possible)
No information available for any classes of bias for the outcome of interest	Unclear risk (or no information)
Classes of bias are judged as having combinations of “moderate”, “serious” or “critical” risks of bias (or other terminology) for the outcome of interest	Summarising judgements other than high/low/unclear may not be intuitively straightforward. A clear rationale should be provided, based on logic (i.e. the criteria should not be arbitrary). See recent Cochrane tools [58, 59, 81] for examples

**Table 6:** Components of a risk of bias assessment that should be reported in the methods section of a systematic review protocol

Item that should be reported in the review protocol	Rationale
1. The tool(s) that will be employed (whether existing, modified, or newly developed by the review team) for assessing each type of study design and the classes of bias and confounding that are covered	A precise description of each risk of bias tool is necessary to enable the overall systematic review methods to be understood, critiqued, and repeated by other researchers, as well as to ensure that the a priori planned approach is followed, to reduce the risk of the review team themselves introducing bias
2. Why and how each tool was selected or developed	Evidence is required that the review team have considered the availability and fitness-for-purpose of existing risk of bias approaches to avoid inappropriate, suboptimal or superseded methods being used
3. The rationale for how the tool assesses the risk of bias	Evidence is required that, as far as can reasonably be inferred, each tool employed has construct validity—that is, it measures the risks of the types of bias that it claims to (rather than measuring factors unrelated to systematic error)
4. How the different classes of bias covered by the tool are defined	Naming and interpretation of bias types is not always consistent in the scientific literature, so an explicit definition of the types of bias to be covered should be provided to avoid misinterpretation
5. Which outcomes the risk of bias assessment will be applied to and whether the process will differ across outcomes	Different outcomes can be subject to different risks of bias or confounders, so it may not always be appropriate to use the same approach across all outcomes
6. The signalling questions that will be used to establish how the review team will classify the risks of bias in a study (if an existing tool is used, this may be cited for such details rather than repeating them)	Signalling questions help to ensure transparency in how the risks of bias in the included studies are identified and classified
7. A copy of the draft template(s) for recording risk of bias assessments (e.g. in an appendix), including the instructions that will be provided to the review team on how to use the tool	Risk of bias judgements are inherently subjective, and it is therefore necessary to provide as complete information as possible on how judgements will be made so the rationale for decisions is clear
8. The number of reviewers who will conduct each assessment and how any disagreements in judgements will be resolved	Single-reviewer assessment of risks of bias could be influenced by implicit bias (i.e. the reviewer’s perspective), so demonstration that the process is not dependent on a single reviewer is required
9. How the risk of bias classification categories used in the tool(s) will be presented and interpreted to inform the data synthesis, both for narrative synthesis and for meta-analysis where applicable (e.g. sensitivity analysis or subgroup analysis)	A priori specification for how risk of bias assessment will inform data synthesis is required to prevent selective inclusion or exclusion of studies in the analysis

**Table 7:** Recommendations for applying results of critical appraisal to inform data synthesis

Recommendation	Rationale
1. All results of risk of bias assessments should inform the data synthesis, whether the synthesis is quantitative (i.e. meta-analysis) and/or narrative	The data synthesis must consider all risks of bias to ensure that the systematic review conclusions can be declared to have high validity, or to have known limitations. This applies whether the data synthesis consists of a quantitative meta-analysis and/or a narrative descriptive synthesis. Both types of synthesis should be clearly structured to demonstrate the impact of study validity on study results
2. Effects of risk of bias should be considered in the data synthesis using sensitivity or subgroup analyses or, if feasible, adjustments in statistical data synthesis models to account for bias	Analysis according to study risk of bias subgroups enables all studies to be included in the data synthesis (including narrative synthesis) and the impact of risk of bias on study outcomes to be explored [55]. This provides a transparent framework for justifying which of the included studies should or should not inform the final review conclusions. Adjustment for some types of confounding such as imbalances in group characteristics may be feasible (e.g. using stratification or statistical modelling such as inverse probability weighting or propensity scoring methods [84]), provided that any threats to validity that cannot be adjusted for are also captured in the data synthesis, in subgroup analyses
3. Do not use numeric summary scores	Numeric scores have several limitations (Box 3) and are not recommended for summarising risk of bias assessments

**Table 8:** Recommendations for reporting the methods and results of critical appraisal in the final systematic review report

Recommendation	Rationale
1. Report the assessment process and criteria employed for conducting risk of bias assessments, including the recording template, signalling questions and any instructions that the review team followed. The protocol may be cited to avoid repeating the details	Clear reporting of all methods employed is essential to facilitate consistent interpretation and ensure reproducibility
2. Report all deviations from the protocol in the systematic review report	To maintain objectivity, transparency and reproducibility of methods, any changes made to the risk of bias assessment should be clearly stated in the final review report, with an explanation of why the changes were necessary
3. Summarise individual risk of bias classes appropriately following the protocol-specified method, reporting details for each class of bias separately in an appendix as well as providing a summary table or chart in the final review report	Both the individual risk of bias classes and a summary of the overall risk of bias conclusion for each study should be reported for each outcome of interest so that the way the summary informs the data synthesis can be communicated clearly whilst maintaining traceability of judgements to the individual contributing studies
4. Provide a concise statement for each risk of bias judgement explaining the rationale for the judgement	Risk of bias judgements are inherently subjective. A clear rationale explaining each judgement is therefore necessary (e.g. presented alongside the categorical judgements in an appendix)
5. Report any limitations of the risk of bias assessment	Any limitations of the process should be concisely stated to ensure that the results are interpreted appropriately

## Data Availability

All data generated or analysed during this study are included in this published article and its supplementary information files.

## Box 1. Challenges in assessing risk of bias in environmental systematic reviews

- Studies included in environmental systematic reviews could have many different designs, each with different risks of bias but identifying the sources of bias requires expert knowledge.
- A complete inventory of the types of study design that could be included in environmental systematic reviews does not exist.
- Many instruments and checklists exist to support identification of risks of bias, but they have been developed mainly in human health and related areas of research and their applicability to environmental studies is unclear.
- The terminology related to assessing risk of bias involves overlapping or synonymous terms including “quality” and “validity” that are not always used consistently, creating opportunities for confusion.
- Existing critical appraisal instruments do not always differentiate between risk of bias and other aspects of study validity or “quality”.
- Published systematic reviews vary widely in whether and how they have assessed and reported risks of bias, and how the assessments were applied to inform their conclusions. This inconsistency serves to create confusion around what should be regarded as good practice.

## Box 3. Limitations of numeric scores for summarising risk of bias

- Numeric scores may imply that different types of bias have equal weight or can be quantified relative to each other.
- Application of numeric scores to categorical data may imply that mathematical operations can be performed on categories (e.g. summing or averaging) that can result in a misleading account of risk of bias for a study.
- A numeric score reported in one systematic review may not have the same meaning as the same score reported in another review.
- Numeric scores cannot capture the direction of bias or its likely magnitude, although this is also a limitation of categorical classification systems.
