# Supplementary material for: Principles and framework for assessing the risk of bias for studies included in comparative quantitative environmental systematic reviews
Source: Environ Evid. Author manuscript; Available in PMC 2024 Jan 23. (PMC10805236; doi:10.1186/s13750-022-00264-0)
Supplement: s2 — Additional file 2. Overview of critical appraisal approaches employed in the ten most recent CEE systematic reviews up to July 2021. [file NIHMS1948588-supplement-s2.docx]

**Additional file 2 Overview of critical appraisal approaches employed in the ten most recent CEE systematic reviews up to July 2021**

**There are 3 tables in this Additional file:**

- Table A: Concordance of recent CEE systematic reviews with the FEAT principles (Focus, Extent, Application, Transparency)
- Table B: Elements of the critical appraisal process reported in recent CEE systematic reviews
- Table C: Overview of critical appraisal approaches in recent CEE systematic reviews (supporting data for Tables A and B)

**Table A Concordance of recent CEE systematic reviews with the FEAT principles**

| **CEE Review** | **FOCUSED?** | **EXTENSIVE?** | **APPLIED?** | **TRANSPARENT?** |
| --- | --- | --- | --- | --- |
| How effective is ‘greening’ of urban areas in reducing human exposure to ground-level ozone concentrations, UV exposure and the ‘urban heat island effect’? An updated systematic review (2021) [1] | YES – All the internal validity criteria appear to reflect potential systematic error in the effect estimate. | NO – A key internal validity domain (attrition bias, i.e. risk of bias due to missing data) was not assessed. | PARTLY – Meta-analysis was rerun without very high risk of bias studies. Risk of bias classes were included in meta-regression but no results presented (stated there was no effect). Inconsistent approach to reporting risk of bias in the narrative synthesis. | PARTLY – Individual bias classes not separable in overall judgement. Inconsistent summary approach for each outcome. Other reporting limitations as noted in Table B below. |
| Are small protected habitat patches within boreal production forests effective in conserving species richness, abundance and community composition? A systematic review (2021) [2] | NO – The stated validity criteria captured internal validity, external validity and also heterogeneity and potential effect modifiers (CEE recommend effect modifiers and heterogeneity are assessed separately from internal validity) | NO – A key internal validity domain (attrition bias, i.e. risk of bias due to missing data) was not assessed. | NO – Critical appraisal not applied to all outcomes. Meta-analysis was re-run excluding high-risk studies for one outcome. Categorisation of low/ medium risk studies not used in the data synthesis. | NO – Lack of clarity around the constructs assessed: text refers to “potential confounding factors” but confounding not explicitly included in the validity criteria. Limited justification of study-level judgements. Other reporting limitations as noted in Table B below. |
| What are the effects of even-aged and uneven-aged forest management on boreal forest biodiversity in Fennoscandia and European Russia? (2021) [3] | NO – The stated validity criteria captured internal validity, external validity and also heterogeneity and potential effect modifiers (CEE recommend effect modifiers and heterogeneity are assessed separately from internal validity) | NO – A key internal validity domain (attrition bias, i.e. risk of bias due to missing data) was not assessed. | NO – Critical appraisal not applied to all outcomes. Meta-analysis was re-run excluding high-risk studies for one outcome. Categorisation of low/ medium risk studies not used in the data synthesis. | NO - Lack of clarity around the constructs assessed – states criteria capture both internal and external validity but also reports overall critical appraisal results as “risk of bias”. Other reporting limitations as noted in Table B below. |
| Effectiveness of struvite precipitation and ammonia stripping for recovery of phosphorus and nitrogen from anaerobic digestate (2020) [4] | PARTLY – The validity criteria mainly capture internal validity but also external validity (is comparison adequately meaningful?, is study generalisable in connection to the review?). | NO – A key internal validity domain (attrition bias, i.e. risk of bias due to missing data) was not assessed. | PARTLY – Studies judged to have flaws in design and which did not meet clarity of reporting criteria were excluded from quantitative synthesis. Studies were included in narrative synthesis provided that they were not judged to have flaws in design. | NO - Limited justification of study-level judgements, and no overall summary judgement provided to integrate individual bias classes. Other reporting limitations as noted in Table B below. |
| Can linear transportation infrastructure verges constitute a habitat and/or a corridor for vertebrates in temperate ecosystems? (2020) [5] | PARTLY – Most of the claimed “risk of bias” items are sources of potential systematic error in outcomes; but criteria also include adequacy of reporting study methods which is not necessarily a source of systematic error. | YES – The key domains of internal validity appear to have been captured, as informed by a stakeholder workshop, although these are not all expressed explicitly as named bias classes. | YES – High risk of bias studies were excluded from the meta-analysis and the influence of low and medium risk studies on effect sizes was compared. Results of studies with low and medium risk were discussed and compared in the narrative synthesis. | PARTLY – Limited justification of study-level judgements. Other reporting limitations as noted in Table B below. |
| What are the relative risks of mortality and injury for fish during downstream passage at hydroelectric dams in temperate regions? (2020) [6] | YES – All the internal validity criteria appear to reflect potential systematic error in outcomes. | NO – A key internal validity domain (attrition bias, i.e. risk of bias due to missing data) was not assessed. | PARTLY – Meta-analysis was rerun excluding low validity studies; high and medium validity studies not analysed separately. | PARTLY – Limited textual justification of study-level judgements. Overall internal validity score in Additional file 5 for each study difficult to trace to component validity sources which are explained in different tables. Other reporting limitations as noted in Table B below. |
| How effective are strategies to control the dissemination of antibiotic resistance in the environment? (2020) [7] | YES – All the internal validity criteria appear to reflect potential systematic error in the effect estimate. | YES – The key domains of internal validity appear to have been captured, although confounding variables listed for individual studies in Additional file 5 are difficult to interpret due to unclear statements. | PARTLY – Meta-analysis sensitivity analyses on effect of study validity were conducted but are not presented in the review report. Narrative synthesis excluded low validity studies and does not distinguish between medium and high validity studies. | NO – Discrepancies between the paper and additional file 4, ambiguous statements in Additional file 5, and inconsistent, suboptimal, tabular layout of extracted data hinders interpretation. No textual rationale provided for study-level judgements. Other reporting limitations as noted in Table B below. |
| Impact of structural habitat modifications in coastal temperate systems on fish recruitment (2019) [8] | NO – Some of the “internal validity” items (“juveniles not separable from adults”, “comparator not relevant”) do not reflect sources of systematic error in outcomes (would be more logical as eligibility criteria). | NO – A key internal validity domain (attrition bias, i.e. risk of bias due to missing data) was not assessed. | PARTLY – Narrative synthesis only, with low validity studies excluded; medium/ high / unclear validity studies not compared explicitly although reader could indirectly make own comparisons based on the data presented | PARTLY - Limited textual justification of study-level judgements. No information on reviewer consensus or whether a process was used for ensuring all threats to validity identified. Other reporting limitations as noted in Table B below. |
| The effectiveness of spawning habitat creation or enhancement for substrate‑spawning temperate fish (2019) [9] | YES – All the internal validity criteria appear to reflect potential systematic error in the effect estimate. | NO – A key internal validity domain (attrition bias, i.e. risk of bias due to missing data) was not assessed. | YES – Low validity studies were excluded from meta-analysis and the remainder were all judged to have medium validity. | PARTLY – Textual description not given for all study-level judgements but rationale for decisions broadly clear. Other reporting limitations as noted in Table B below. |
| To what extent do mesophotic coral ecosystems and shallow reefs share species of conservation interest? (2018) [10] | NO – One of the “internal validity” items (study being a taxonomic key) does not reflect a source of systematic error in outcomes (would be more logical as an eligibility criterion). | NO – Key internal validity domains (risk of selection bias, risk of bias due to missing data) were not assessed. | PARTLY – Meta-analysis sensitivity analysis on high-validity studies versus all studies, but validity not considered in the narrative synthesis. | NO – Limited textual justification of study-level judgements. Discrepancy in data between paper and Additional file 2. Other reporting limitations as noted in Table B below. |

**Table B Elements of the critical appraisal process reported in recent CEE systematic reviews**

| **CEE Review** | **Process for identifying all key threats to internal validity (i.e. risks of bias and confounding)** | **Piloting of the critical appraisal criteria and process** | **Number of reviewers conducting critical appraisal (after any pilot phase)** | **Reviewer agreement or reliability on critical appraisal judgements** |
| --- | --- | --- | --- | --- |
| How effective is ‘greening’ of urban areas in reducing human exposure to ground-level ozone concentrations, UV exposure and the ‘urban heat island effect’? (2021) [1] | Not reported, other than based on experience with a previous version of the SR. | Two reviewers assessed a random 10% sample of studies from the initial (2016) search. There was minor inconsistency in interpretation of one element of study design which was clarified. | One reviewer (for the initial 2016 and updated 2018 searches). A second reviewer independently checked 10/71 studies from the 2018 search and discussed any uncertainties raised by the first reviewer. | Stated no systematic differences between reviewers were identified (no data presented). |
| Are small protected habitat patches within boreal production forests effective in conserving species richness, abundance and community composition? (2021) [2] | Not reported, other than critical appraisal was based on factors considered important by the authors of the review. | Not reported | Two reviewers | Not reported |
| What are the effects of even-aged and uneven-aged forest management on boreal forest biodiversity in Fennoscandia and European Russia? (2021) [3] | Not reported, other than critical appraisal was based on factors considered important by the authors of the review. | Initial consistency check on 100 studies by 2 reviewers. Decisions differed in less than 5% of the studies, and related to appraisal criteria on study design and sampling, which were clarified when decisions were discussed by to improve consistency. | One reviewer (uncertain cases were discussed and the risk of bias was determined jointly by the research group members; but not stated how many cases were uncertain). | Not reported |
| Effectiveness of struvite precipitation and ammonia stripping for recovery of phosphorus and nitrogen from anaerobic digestate (2020) [4] | Stated that the detailed criteria for the study validity assessment of eligible studies (i.e. critical appraisal tool) was developed and trialled during the review process in several meetings with subject experts, but no details reported. | Tool was piloted on 10% of studies by the entire team; no other details reported. | Two reviewers | Not reported |
| Can linear transportation infrastructure verges constitute a habitat and/or a corridor for vertebrates in temperate ecosystems? (2020) [5] | 1-day stakeholder workshop with 8 external experts and 7 members of review team. Discussed the gold standard protocol of an ideal study that would answer the primary question with  unlimited resources. | Not reported | One reviewer (uncertain cases were assessed by a second reviewer but not stated how many were uncertain). Disagreements (number not reported) were referred to a third reviewer if necessary. | Not reported |
| What are the relative risks of mortality and injury for fish during downstream passage at hydroelectric dams in temperate regions? (2020) [6] | The framework used to assess study validity was reviewed by the Advisory Team to ensure that it  accurately reflected the characteristics of a well-designed study. | Three reviewers initially assessed 7.8% of studies and where necessary the validity assessment was refined to improve clarity in coding. | One reviewer (not reported how any uncertain cases were handled). | Not reported |
| How effective are strategies to control the dissemination of antibiotic resistance in the environment? (2020) [7] | Not reported other than sources of bias were determined through discussion with the review team. | Not reported | Not reported. Stated that the whole review team contributed to study validity assessment and all decisions were double-checked by the  project manager. Disagreements were discussed during  working meetings until an agreement was reached (no data presented). | Not reported |
| Impact of structural habitat modifications in coastal temperate systems on fish recruitment (2019) [8] | Not reported | 10% of studies were initially appraised by the entire review team to check for appraisal consistency, and all disagreements  were discussed and the criteria further refined. | Two reviewers. Final  decisions regarding doubtful cases were taken by the  whole review team (not reported how many cases were doubtful). | Not reported |
| The effectiveness of spawning habitat creation or enhancement for substrate‑spawning temperate fish (2019) [9] | The framework used to assess study validity was reviewed by the Advisory Team to ensure that it accurately reflected  the characteristics of an ideal study, regardless of resources or experimental/ field restrictions. | 6/94 studies (9.4%) were initially checked for consistency by 2 reviewers. Discrepancies were discussed and, when necessary, refinements to the meta-data extraction and quality assessment sheets were made to improve clarity on coding. | One reviewer. Any uncertainty was discussed with a second reviewer, but not stated how many cases were uncertain. | Not reported |
| To what extent do mesophotic coral ecosystems and shallow reefs share species of conservation interest? (2018) [10] | Not reported | Not reported | Not reported | Not reported |

**Table C Overview of critical appraisal approaches in recent CEE systematic reviews (supporting data for Tables A and B)**

| **CEE Review** | **Focus of critical appraisal** | **Critical appraisal output** | **Process to ensure all key threats to internal validity identified** | **Piloting & reviewer consensus** | **How critical appraisal informed data synthesis** |
| --- | --- | --- | --- | --- | --- |
| How effective is ‘greening’ of urban areas in reducing human exposure to ground-level ozone concentrations, UV exposure and the ‘urban heat island effect’? (2021) [1] | Selection bias (defined)  Detection bias (defined)  Performance bias (defined)  Confounding (not defined)  Not explicitly covered:  Attrition bias  Outcome reporting bias | Risk of bias (also called validity). Defined by criteria which integrate the individual bias & confounding domains to give an overall judgement (page 6):  Very high (defined)  High (defined)  Moderate (defined)  Low (defined)  Textual rationale for each judgement provided (Additional file 1) | Not stated, other than based on experience with a previous version of the SR (page 6). | 2 reviewers assessed a random 10% sample of studies from the initial (2016) search. There was minor inconsistency in interpretation of one element of study design which was clarified. 1 reviewer (for the initial 2016 and updated 2018 searches). A 2^nd^ reviewer independently checked 10/71 studies from the 2018 search and discussed any uncertainties raised by the 1^st^ reviewer. Stated no systematic differences between reviewers were identified (no data presented). | Meta-analysis was run with and without the very high risk of bias studies page 9). Also states RoB classes were converted to numeric scores and included in meta-regression analyses (page 16). No quantitative results of these analyses are provided (but stated there was no effect). Risk of bias assessments do not consistently inform narrative synthesis conclusions across outcomes. |
| Are small protected habitat patches within boreal production forests effective in conserving species richness, abundance and community composition? (2021) [2] | Described as “risk of bias” but based on ad hoc criteria for study design, sampling approach (sample size, sampling method, treatment matching) heterogeneity & potential effect modifiers & statistical data analysis methods. Not formalised as classes of bias. Text refers to “potential confounding factors” but confounding not explicitly included in criteria. Attrition (missing data) not explicitly considered. | Studies given an overall classification as:  Low risk (defined)  Medium risk (defined)  High risk (defined)  Unclear risk  Definitions do not map to specific classes of bias  Arbitrarily judged that studies in “low” and “medium” categories had “sufficient quality” to provide a reliable evidence base for quantitative synthesis.  Textual rationale not provided for each judgement except basic standard statement for high risk studies (“effect modifiers not considered” or “comparing rest of studies using different method”) (Additional file 7) | Stated only that critical appraisal was based on factors considered by the authors of the review to be key variables related to generalisability and reliability of study  findings | Piloting of criteria not reported.  2 reviewers assessed studies at same time as data extraction; consensus not reported | Meta-analysis was re-run excluding high-risk studies (page 16; Additional file 10) but only for one outcome (volume of deadwood; not assessed for species richness or abundance). Categorisation of low/medium risk studies not used (none were judged unclear) |
| What are the effects of even-aged and uneven-aged forest management on boreal forest biodiversity in Fennoscandia and European Russia? (2021) [3] | Stated that validity criteria captured both internal and external validity but also studies were categorised as low, medium or high risk of bias. The ad hoc validity criteria were design, sampling approach (sample size, sampling method, treatment matching) heterogeneity & potential effect modifiers & statistical data analysis methods. Not formalised as classes of bias. Text refers to “potential confounding factors” but confounding not explicitly included in criteria. Attrition (missing data) not explicitly considered. | Studies given an overall critical appraisal classification as:  Low risk (defined)  Medium risk (defined)  High risk (defined)  Unclear risk  Definitions do not map to specific bias classes  Arbitrarily judged that studies in “low” and “medium” categories had “sufficient quality” to provide a reliable evidence base for quantitative synthesis.  Textual rationale provided for each judgement but in some cases only as basic statements such as “observational study” whereas more detailed statements given in other cases for the same validity class (Additional files 5 & 6). Observational studies were classed as medium risk as the researcher had no control over the exposure. High risk studies had “unsuitable analysis methods”. | Stated only that critical appraisal was based on factors considered by the authors of the review to be key variables related to generalisability and reliability of study  findings | Initial consistency check with with 100 studies by 2 reviewers. Stated there was a high agreement on which articles to include as decisions differed in less than 5% of the studies (calculated as different decisions per 100 studies). The differences related to appraisal criteria on study design and sampling, which were clarified when decisions were discussed to improve consistency. Any uncertain decisions during the critical appraisal were discussed jointly by the research group members. | Meta-analysis was re-run excluding high-risk studies (page 18; Additional file 8) but only for overall abundance outcome (not abundance by taxonomic subgroups or species richness). Categorisation of low/medium risk studies not used in data synthesis. |
| Effectiveness of struvite precipitation and ammonia stripping for recovery of phosphorus and nitrogen from anaerobic digestate (2020) [4] | Ad hoc tool developed to assess internal and external validity. Covered: (1) study set up and design flaws (due to calculation errors, invalid outcome measurements or failure to control for the effect of additional competing interventions) and (2) susceptibility to bias (in the form of reporting bias, i.e. selective reporting of study findings). Attrition (missing data) not explicitly considered. | All the studies included in the quantitative synthesis were judged to be similar in quality, so no distinction was made between different quality studies, and no weighting was done.  Additional file 5 presents Y/N answers for (1) any confounding factors? (2) confounding factors controlled for? (3) Is study generalisable in connection to the review? (4) Is comparison adequately meaningful? (5) Is sampling frequency appropriate? (6) Is spatial replication performed in an appropriate way? Brief textual rationale for each judgement is given for one outcome (ammonia stripping) but not other outcome (struvite precipitation). No overall validity summary judgement provided to integrate these factors. | Stated only that the detailed criteria for the study validity assessment of eligible studies (i.e. critical appraisal tool) was  developed and trialled during the review process in several meetings with subject experts. | Tool was piloted on 10% of studies by the entire team.  Each study was assessed by two independent reviewers. Final decisions regarding doubtful cases were taken by the whole review team | Studies judged to  have flaws in design and setup or reporting bias were excluded from the narrative and quantitative synthesis. Remaining studies were assessed for clarity of reporting key information and if unclear on 2 or more reporting domains were classified as ‘unclear’ and excluded from quantitative synthesis but included in the narrative synthesis. Studies passing both appraisal steps were  included in the quantitative synthesis |
| Can linear transportation infrastructure verges constitute a habitat and/or a corridor for vertebrates in temperate ecosystems? (2020) [5] | Combination of internal validity and adequacy of reporting, described collectively as “risk of bias”, using criteria developed specifically for this review.  Low “risk of bias” assumed if a study was not classified as high or medium risk.  Critical appraisal was conducted separately for each of 6 review questions. | High risk of bias if:  No replications; inadequate methodology; strongly insufficient method description; major confounding factors (e.g. strong difference in sampling effort between treatment and control).  Medium risk of bias if:  sample location selection not transparent and systematic (i.e. randomization, fixed distances, grids); CI and BA study designs used (as opposed to BACI designs) for 2 specific review questions; no true spatial replication of study; attrition bias (difference in loss of samples between control & treatment); method description slightly insufficient (missing details did not challenge understanding of the methods.  Textual rationale provided for each judgement very limited (e.g. “confounding factors”, “inappropriate format”) | 1-day stakeholder workshop with 8 external experts and 7 members of review team. Discussed gold standard protocol  of an ideal study that would answer the primary question with  unlimited resources. | Piloting of criteria not reported.  One reviewer assessed each study, with uncertain cases (number not reported) assessed by a second reviewer. Disagreements (number not reported) were referred to a 3^rd^ reviewer if necessary. | High “risk of bias” studies excluded from review. For low and medium risk studies the “risk of bias” was correlated with the type of exposure (e.g. all studies on powerlines had a low risk of bias whereas all studies on railways had a medium risk of bias). Stated there was no effect of low or medium risk of bias on effect sizes (page 22).  Results of studies with low and medium “risk of bias” were appropriately discussed and compared in the narrative synthesis. |
| What are the relative risks of mortality and injury for fish during downstream passage at hydroelectric dams in temperate regions? (2020) [6] | Internal validity tool developed ad hoc for this review, capturing selection, performance and assessment bias (external validity also assessed separately).  NB some aspects of quality not related to systematic error also captured, e.g. sufficiency of reporting.  Attrition (missing data) not explicitly considered. | Defined criteria for high, medium and low validity map to selection, performance, and assessment biases as stated in Table 3 & Additional file 6.  Assessment bias criteria included sample size (arbitrary cutoff, high validity if >100 fish; medium validity if >50<100 fish). Outcome metric had to be appropriate for review (arguably an eligibility criterion, not a systematic error).  Selection bias was assessed based on study design (irrespective whether randomised), and comparability and homogeneity of treatment and control samples) (NB no criteria for medium/low given for study design in Table 3 or Additional file 6).  Low validity could be assigned if insufficient information was reported (arguably a reporting issue, not systematic error).  Study was scored “low validity” if judged to be of low validity on one or more criteria. Scored “high validity” if judged to have high validity on all criteria. Scored “medium validity” if no criteria were judged low validity criteria.  Generic, not study-specific, textual justifications for judgements provided in Additional file 5 but don’t name any study-specific confounders. | Stated only that the framework used to assess study validity was reviewed by the Advisory Team to ensure that it  accurately reflected the characteristics of a well-designed  study | 3 reviewers initially assessed 7.8% of studies and where necessary the validity assessment was refined to improve clarity in coding. One reviewer assessed validity for the remaining 92.2% of studies. | No study was excluded based study validity assessments. However, a sensitivity analysis was carried out by re-running meta-analysis excluding low validity studies (stated that high and medium validity were not analysed separately as there were only 2 criteria for which a medium score could be applied, as shown in Table 4; NB 12% of studies had medium validity; page 20).  The main data synthesis results include consideration of study validity (all studies versus high + medium validity studies) in relation to outcomes. However, study validity is not considered in analyses of the effects of moderators. |
| How effective are strategies to control the dissemination of antibiotic resistance in the environment? (2020) [7] | Internal validity classes assessed (defined in Table 3):   - Confounding bias - Selection bias - Performance bias - Detection bias - Attrition bias - Reporting bias | Validity was classified using various combinations of the following depending on the study type and management strategy (Additional file 4):   - High - Definitely high - Probably high - Probably high or medium - Probably low - Definitely low - Low   “Probably” and “definitely” were used where necessary to avoid all studies being classed as having medium validity)  A textual rationale is not provided for each validity judgement. | Stated only that sources of  bias were determined through discussion with the review  team and biases were defined using the catalogue of biases  (https://catalogofbias.org/biases/) | Piloting of criteria not reported.  Stated that the “whole review team contributed to study validity assessment” but no other information given. | Stated that sensitivity analyses on the effect of study validity were conducted in the quantitative synthesis and no major effect was identified. However, these analyses are not clear (“confidence levels” rather than validity are reported in sensitivity analyses in Additional file 7).  Low validity (or probably low and definitely low validity) studies were excluded from narrative synthesis. The narrative synthesis does not distinguish the medium and high validity studies or the probably high and definitely high validity studies. |
| Impact of structural habitat modifications in coastal temperate systems on fish recruitment (2019) [8] | Assessed internal validity using an approach similar to that used by other EViEM reviews. Not framed as specific classes of bias, although capturing some aspects of these.  Attrition (missing data) not explicitly considered. | Low validity criteria:  No replication; exposure and comparator sites not matched; severely confounding factors present (e.g. additional  treatments carried out or different sampling method between  exposure and control sites); juvenile data not separable from adult data.  Medium validity criteria:  BA study design (not CI or BACI); suboptimal sample replication, e.g. pseudoreplication or unbalanced sampling design.  Unclear validity criteria:  Poor methodological description or exposure difficult to interpret.  High validity:  If none of the above factors applied.  Generic, not study-specific, textual justifications for judgements provided but don’t name any study-specific confounders. | None stated | The whole review team initially assessed 10% of studies to check for consistency, discuss disagreements and refine the criteria. Two reviewers then appraised each study. Final decisions regarding doubtful cases were taken by the whole review team (not reported how many cases were doubtful). | Studies with high, medium and unclear validity were included  in the narrative synthesis, whereas studies with low validity (i.e. high susceptibility to bias) were excluded. (No quantitative synthesis was conducted).  Medium/high/unclear validity study results were not compared explicitly in the synthesis (although the reader could deduce indirectly which results relate to these validity classes based on the information presented (Table 2). |
| The effectiveness of spawning habitat creation or enhancement for substrate‑spawning temperate fish (2019) [9] | Ad hoc tool was developed to assess internal validity criteria (defined in Table 1):  Selection bias (study design, replication, matching of intervention  & comparator sites)  Assessment bias (measured outcome quantitative, semi-quantitative or approximate)  Performance bias (other confounding environmental factors).  Attrition (missing data) not explicitly considered. | Validity classified as  High  Medium  Low  If the study did not score at least one low or all high for any of the criteria, it was assigned an overall ‘medium’ validity. Studies that scored only high for all of the criteria were assigned an overall ‘high’ validity.  Appropriately detailed textual description provided to justify study-specific “confounding factors” judgement in Additional file 3. Rationale for other judgements based on generic wording but broadly clear. | Stated that critical appraisal framework was based on a recommendation by Bilotta et al [11] and adapted to incorporate components specific to studies that answer the primary review question. The framework used to assess study validity was reviewed by the Advisory Team to ensure that it accurately reflected the characteristics of an ideal study, regardless of resources or experimental/field restrictions. | Initial consistency check done on 6/64 articles (9.4%) by 2 reviewers, then all remaining articles assessed by one reviewer, with any uncertainty discussed with a second reviewer. Consensus not reported. | Low validity studies were excluded from meta-analysis; all included studies were judged as having medium validity. Adjustments were made in the meta-analysis to account for cases of (presumed or actual) pseudo-replication (method in Additional file 5).  Narrative synthesis included all studies regardless of validity (but did not report quantitative outcome measures) |
| To what extent do mesophotic coral ecosystems and shallow reefs share species of conservation interest? (2018) [10] | “internal validity” criteria were:   - >10 species identified within a taxon (to limit undue influence of single species on community overlap values) - The study is not a regional taxonomic key - Even sampling is reported across depths   Selection & attrition biases not explicitly considered. | Studies passing all three criteria (left) were included in a “high validity” subgroup.  No other validity classes were defined.  Short generic, not study-specific, textual justifications for judgements provided (Additional file 2) but don’t name any study-specific confounders. | None reported. Stated only that with respect to internal validity, the authors’ primary concern with mesophotic research is that sampling effort may decline with increasing depth which can lead to an underestimate of deeper species. | No information reported | Meta-analysis was repeated on the subset of high-validity studies  Brief narrative synthesis (e.g. Figure 3 which relates to species depth distribution) does not consider the study validity classification (i.e. Figure 3 may include studies that had uneven depth sampling) |

**References**

1. Knight T, Price S, Bowler D, Hookway A, King S, Konno K, et al. How effective is ‘greening’ of urban areas in reducing human exposure to ground-level ozone concentrations, UV exposure and the ‘urban heat island effect’? An updated systematic review. Environmental Evidence. 2021;10:12:1-38.

2. Häkkilä M, Johansson A, Sandgren T, Uusitalo A, Mönkkönen M, Puttonen P, et al. Are small protected habitat patches within boreal production forests effective in conserving species richness, abundance and community composition? A systematic review. Environmental Evidence. 2021;10:2:1-20.

3. Savilaakso S, Johansson A, Häkkilä M, Uusitalo A, Sandgren T, Mönkkönen M, et al. What are the effects of even-aged and uneven-aged forest management on boreal forest biodiversity in Fennoscandia and European Russia? A systematic review. Environmental Evidence. 2021;10:1:1-38.

4. Lorick D, Macura B, Ahlström M, Grimvall A, Harder R. Effectiveness of struvite precipitation and ammonia stripping for recovery of phosphorus and nitrogen from anaerobic digestate: a systematic review. Environmental Evidence. 2020;9:27:1-20.

5. Ouédraogo D-Y, Villemey A, Vanpeene S, Coulon A, Azambourg V, Hulard M, et al. Can linear transportation infrastructure verges constitute a habitat and/or a corridor for vertebrates in temperate ecosystems? A systematic review. Environmental Evidence. 2020;9:13:1-34.

6. Algera DA, Rytwinski T, Taylor JJ, Bennett JR, Smokorowski KE, Harrison PM, et al. What are the relative risks of mortality and injury for fish during downstream passage at hydroelectric dams in temperate regions? A systematic review. Environmental Evidence. 2020;9:3:1-36.

7. Goulas A, Belhadi D, Descamps A, Andremont A, Benoit P, Courtois S, et al. How effective are strategies to control the dissemination of antibiotic resistance in the environment? A systematic review. Environmental Evidence. 2020;9:4:1-32.

8. Macura B, Byström P, Airoldi L, Eriksson BK, Rudstam L, Støttrup JG. Impact of structural habitat modifications in coastal temperate systems on fish recruitment: a systematic review. Environmental Evidence. 2019;8:14:1-22.

9. Taylor JJ, Rytwinski T, Bennett JR, Smokorowski KE, Lapointe NWR, Janusz R, et al. The effectiveness of spawning habitat creation or enhancement for substrate‑spawning temperate fish: a systematic review. Environmental Evidence. 2019;8:19:1-31.

10. Laverick JH, Piango S, Andradi-Brown DA, Exton DA, Bongaerts P, Bridge TCL, et al. To what extent do mesophotic coral ecosystems and shallow reefs share species of conservation interest? A systematic review. Environmental Evidence. 2018;7:15:1-15.

11. Bilotta G, Milner A, Boyd I. Quality assessment tools for evidence from environmental science. Environmental Evidence. 2014;3:14:1-14.

____________________________________________________________________________________________________________________________

This Additional file is part of the article *Principles and framework for assessing the risk of bias for studies included in comparative quantitative environmental systematic reviews.* Environmental Evidence journal 2022.
