# Supplementary material for: Principles and framework for assessing the risk of bias for studies included in comparative quantitative environmental systematic reviews
Source: Environ Evid. Author manuscript; Available in PMC 2024 Jan 23. (PMC10805236; doi:10.1186/s13750-022-00264-0)
Supplement: s3 — Additional file 3. Examples of study designs that have been included in environmental systematic reviews (SR) and meta-analyses (MA) addressing PECO or PICO-type questions. [file NIHMS1948588-supplement-s3.docx]

**Additional file 3 Examples of study designs that have been included in environmental systematic reviews (SR) and meta-analyses (MA) addressing PECO or PICO-type questions**

| **Type of study design** | **Description** | **Examples** | **Source of examples** |
| --- | --- | --- | --- |
| **Controlled before-after control- intervention (BACI) study**  (randomized or non-randomized) | A study in which differences in outcome(s) before and after an intervention or exposure of interest is applied are compared between intervention/ exposure group(s) and non-intervention/ non-exposure group(s) | - Randomized: Effect on litter decomposition of transgenic herbicide-tolerant maize compared to conventional maize in randomized replicated field plots in Canada (1) | SR (2) |
|  |  | - Non-randomized: Invasive weed seed bank compared before and after three different roadside verge mowing regimes in Austria (3) | SR (4) |
|  |  | - Non-randomized: Vegetation diversity compared before and after three different types of meadow management in Finland (5) | SR (6) |
|  |  | - Non-randomized: Lake water quality compared before and after manipulation of fish biomass (7) | SR (8) |
| **Control-intervention (CI) study**  (randomized or non-randomized) | A study in which the focus of comparison is the difference in outcome(s) between intervention/ exposure group(s) and concurrent control group(s) (or between different concurrent intervention/ exposure groups) | - Randomized: Effect on an insect pest and its natural enemies of transgenic insect-resistant maize compared to conventional maize in randomized replicated field plots in the USA (9) | MA (10) |
|  |  | - Non-randomized: Effects on biodiversity compared after manipulating deer abundance in replicated plots in the Midwest USA (11) and on islands in Canada (12) | SR (6) |
|  |  | - Non-randomized: Crop yields compared between a maize rotation with green cover crop (intervention) and a traditional maize rotation (control), at several community sites in two Mexican forest reserve areas (13) | SR (14) |
|  |  | - Non-randomized: Insect abundance compared in eight alternating plots with herbicide treatment (intervention) and without herbicide treatment (control) situated along one edge of a cereal field in England (15) | MA (16) |
| **Before-after (BA) study** | A study in which the focus of comparison is the difference in outcome(s) before and after an intervention or exposure of interest is applied | - Soil invertebrates monitored before and after a forest burn at a single site in the USA (17) | SR (18) |
| **Case-control study** | Exposure/intervention sites are compared to control sites without the exposure/ intervention. May include one or more pairs of cases and controls. | - Comparison of species diversity between community-managed and government-managed mountain forests in Nepal (19) | SR (20) |
|  |  | - Psychological health compared using post-flood phone interviews between previously flooded households (**cases**) and not-flooded households (**controls**) from the same locality in England (21) | SR (22) |
| **Temporal monitoring study (may be called prospective longitudinal or cohort study). Includes interrupted time series analyses.** | Monitoring based on data collected prospectively or retrospectively over a number of years. May include analyses of association, correlation or regression to explain trends or investigate temporality (sequence of events). | - Soil properties sampled over 30 years in replicated small conventional-tillage and no-tillage plots at a single site in the USA (23) | SR (24) |
|  |  | - Birth outcomes monitored in women who were pregnant during, or became pregnant immediately after, natural disasters (hurricanes) in the USA (25) | SR (22) |
|  |  | - Time series of atmospheric organic pollutant deposition estimated using Tibetan snow cores (26) - Time series of organic pollutant body burden obtained from archived Norwegian human tissue samples (27) - Organic pollutants sampled using historic data from single sites, e.g. drinking water in Uppsala, Sweden (28), sediments of Lake Ontario, Canada (29) and sediments of Lake Chaohu, China (30) | SR (31) |
| **Spatial monitoring study** | Multi-site sampling in one or few years | - Organic pollutants sampled in the atmosphere at a range of globally distributed sites (32) | SR (31) |
| **Spatial and temporal monitoring study** | Multi-year sampling at multiple sites. | - Soil properties in arable crop rotations compared over 18-25 years between three tillage interventions in unreplicated small plots, repeated at four sites in Germany (33) | SR (24) |
| **Cross-sectional study survey of prevalence, occurrence, or characteristics** | A study that assesses the characteristics of a system at a single point in time. | - Survey to determine occurrence and properties of algal beds in a Brazilian archipelago (34) - Survey of changes in the diversity and trophic group structure of Caribbean coral reef fish populations across a depth gradient (35) | SR (36) |
| **Cross-sectional survey of people’s attitudes, opinions or beliefs (i.e. including interview, questionnaire or focus group)** | A cross-sectional study that requires human responses. Potentially at risk of recall bias. May be the main study design, but often included within another type of study design. | - Survey of attitudes, beliefs, knowledge and behaviour relating to butterfly farming in butterfly farmer (**cases**) and **control** groups (authors referred to it as a (quasi-experimental’ design) (37) - Survey of attitudes, beliefs, knowledge and behaviour relating to efficient stove use **before and after** a monkey protection social marketing campaign in a Chinese nature reserve (38) - Questionnaires and focus groups to investigate seaweed farming impact over time on fisher numbers in Philippine villages (39) | SR (14) |
| **Case study or descriptive study** | A study that is applicable to a single population or system at a specific time point | - Case studies of fish biomanipulation in three Danish lakes (40) | SR (8) |

**References**

1. Powell JR, Levy-Booth DJ, Gulden RH, Asbil WL, Campbell RG, Dunfield KE, et al. Effects of genetically modified, herbicide-tolerant crops and their management on soil food web properties and crop litter decomposition. Journal of Applied Ecology. 2009;46:388-96.

2. Knox O, Hall C, McVittie A, Walker R, Knight B. A systematic review of the environmental impacts of GM crop cultivation as reported from 2006 to 2011. Food and Nutrition Sciences. 2013;4:28-44.

3. Milakovic I, Karrer G. The influence of mowing regime on the soil seed bank of the invasive plant Ambrosia artemisiifolia L. NeoBiota. 2016;28:39-49.

4. Jakobsson S, Bernes C, Bullock JM, Verheyen K, Lindborg R. How does roadside vegetation management affect the diversity of vascular plants and invertebrates? A systematic review. Environmental Evidence. 2018;7:17:1-13.

5. Kotiluoto R. Vegetation changes in restored semi-natural meadows in the Turku Archipelago of SW Finland. Plant Ecology. 1998;136:53-67.

6. Bernes C, Macura B, Jonsson BG, Junninen K, Müller J, Sandström J, et al. Manipulating ungulate herbivory in temperate and boreal forests: effects on vegatetion and invertebrates. Environmental Evidence. 2018;7:13:1-32.

7. Hanson MA, Butler MG. Responses of plankton, turbidity, and macrophytes to biomanipulation in a shallow prairie lake. Canadian Journal of Fisheries and Aquatic Sciences. 1994;51(5):1180-8.

8. Bernes C, Carpenter SR, Gårdmark A, P. L, Persson L SC, Speed JDM, et al. What is the influence of a reduction of planktivorous and benthivorous fish on water quality in temperate eutrophic lakes? A systematic review. Environmental Evidence. 2015;4:7:1-28.

9. Orr DB, Landis DA. Oviposition of the European corn borer (Lepidoptera: Pyralidae) and impact of natural enemy populations in transgenic versus isogenic corn. Journal of Economic Entomology 1997;90(4):905-9.

10. Marvier M, McCreedy C, Regetz J, Karieva P. A meta-analysis of effects of Bt cotton and maize on nontarget invertebrates. Science. 2007;316(5830):1475-7.

11. Shelton AL, Henning JA, Schultz P, Clay K. Effects of abundant white-tailed deer on vegetation, animals, mycorrhizal fungi, and soils. Forest Ecology and Management. 2014;320:39-49.

12. Chollett S, Padié S, Stockton S, Allombert S, Gaston AJ, Martin J-L. Positive plant and bird diversity response to experimental deer population reduction after decades of uncontrolled browsing. Diversity and Distributions. 2016;22:274-87.

13. Eastmond A, Faust B. Farmers, fires, and forests: a green alternative to shifting cultivation for conservation of the Maya forest? Landscape and Urban Planning. 2006;74(3-4):267-84.

14. Roe D, Booker F, Day M, Zhou W, Allebone-Webb S, Hill N, et al. Are alternative livelihood projects effective at reducing local threats to specified elements of biodiversity and/or improving or maintaining the conservation status of those elements? Environmental Evidence. 2015;4:22:1-22.

15. Chiverton PA, Sotherton NW. The effects on beneficial arthropods of the exclusion of herbicides from cereal crop edges. Journal of Applied Ecology. 1991;28(3):1027-39.

16. Frampton G, Dorne J. The effects on terrestrial invertebrates of reducing pesticide inputs in arable crop edges: A meta-analysis. Journal of Applied Ecology. 2007;44:362-73.

17. Crossley DA, Hansen RA, Lamoncha KL. Response of forest floor microarthropods to a forest regeneration burn at Wine Spring watershed (Southern Appalachians). First Biennial North American Forest Ecology Workshop, JUne 22-24, 1997. 1997.

18. Eales J, Haddaway NR, Bernes C, Cooke SJ, Jonsson BG, Kouki J, et al. What is the effect of prescribed burning in temperate and boreal forest on biodiversity, beyond pyrophilous and saproxylic species? A systematic review. Environmental Evidence. 2018;7:19:1-33.

19. Måren IE, Bhattarai KR, Chaudhary RP. Forest ecosystem services and biodiversity in contrasting Himalayan forest management systems. Environmental Conservation. 2013;41(1):1-11.

20. Ojanen M, Zhou W, Miller D, Nieto S, Mshale B, Petrokofsky G. What are the environmental impacts of property rights regimes in forests, fisheries and rangelands? Environmental Evidence. 2017;6:12:1-23.

21. Reacher M, McKenzie K, Lane C, Nichols T, Kedge I, Iversen A, et al. Health impacts of flooding in Lewes: a comparison of reported gastrointestinal and other illness and mental health in flooded and non-flooded households. Communicable Disease and Public Health. 2004;7:39-46.

22. Alderman K, Turner LR, Tong S. Floods and human health: a systematic review. Environment International. 2012;47:37-47.

23. Devine S, Markewitz D, Hendrix P, Coleman D. Soil aggregates and associated organic matter under conventional tillage, no-tillage, and forest succession after three decades. PLoS One. 2014;9(1): e84988:1-12.

24. Haddaway NR, Hedlund K, Jackson LE, Kätterer T, Lugato E, Thomsen IK, et al. How does tillage intensity affect soil organic carbon? A systematic review. Environmental Evidence. 2017;6:30:1-48.

25. Xiong X, Harville E, Mattison D, Elkind-Hirsch K, Pridjian G, Buekens P. Exposure to Hurricane Katrina, post-traumatic stress disorder and birth outcomes. The American Journal of the Medical Sciences. 2008;336:111-5.

26. Wang X, Halsall C, Codling G, Xie Z, Xu B, Zhao Z, et al. Accumulation of perfluoroalkyl compounds in Tibetan mountain snow: Temporal patterns from 1980 to 2010. Environmental Science and Technology. 2014;48:173-81.

27. Haug LS, Thomsen C, G B. Time trends and the influence of age and gender on serum concentrations of perfluorinated compounds in archived human samples. Environmental Science and Technology. 2009;43(6):2131-6.

28. Gyllenhammar I, Berger U, Sundström M, McCleaf P, Eurén K, Eriksson S, et al. Influence of contaminated drinking water on perfluoroalkyl acid levels in human serum - a case study from Uppsala, Sweden. Environmental Research. 2015;140:673-83.

29. Yeung LW, De Silva AO, Loi EI, Marvin CH, Taniyasu S, Yamashita N, et al. Perfluoroalkyl substances and extractable organic fluorine in surface sediments and cores from Lake Ontario. Environment International. 2013;59:389-97.

30. Qi Y, Hu S, Huo S, Xi B, Zhang J, Wang X. Spatial distribution and historical deposition behaviors of perfluoroalkyl substances (PFASs) in sediments of Lake Chaohu, a shallow eutrophic lake in Eastern China. Ecological Indicators. 2015;57:1-10.

31. Land M, de Wit CA, Bignert A, Cousins IT, Herzke D, Johansson JH, et al. What is the effect of phasing out long-chain per- and polyfluoroalkyl substances on the concentrations of perfluoroalkyl acids and their precursors in the environment? A systematic review. Environmental Evidence. 2018;7:4:1-32.

32. Gawor A, Shunthirasingham C, Hayward SJ, Lei YD, Gouin T, Mmereki BT, et al. Neutral polyfluoroalkyl substances in the global Atmosphere. Environmental Science - Processes and Impacts. 2014;16:404-13.

33. Andruschkewitsch R, Geisseler D, Koch H-J, Ludwig B. Effects of tillage on contents of organic carbon, nitrogen, water-stable aggregates and light fraction of four different long-term trials. Geoderma. 2013;192:368-77.

34. Amado-Filho GM, Pereira-Filho GH, Bahia RG, Abrantes DP, Veras PC, Matheus Z. Occurrence and distribution of rhodolith beds on the Fernando de Noronha Archipelago of Brazil. Aquatic Botany. 2012;101:41-5.

35. Andradi-Brown DA, Gress E, Wright G, Exton DA, Rogers AD. Reef fish community biomass and trophic structure changes across shallow to upper-mesophotic reefs in the Mesoamerican Barrier Reef, Caribbean. PLoS One. 2016;11(6): e0156641:1-19.

36. Laverick JH, Piango S, Andradi-Brown DA, Exton DA, Bongaerts P, Bridge TCL, et al. To what extent do mesophotic coral ecosystems and shallow reefs share species of conservation interest? A systematic review. Environmental Evidence. 2018;7:15:1-15.

37. Morgan-Brown T, Jaconson SK, Wald K, Childs B. Quantitative assessment of a Tanzanian integrated conservation and development project involving butterfly farming. Conservation Biology. 2010;24:563-72.

38. DeWan A, Green K, Li X, Hayden D. Using social marketing tools to increase fuel-efficient stove adoption for conservation of the golden snub-nosed monkey , Gansu Province, China. Conservation Evidence. 2013;2013:32-6.

39. Hill NAO, Rowcliffe JM, Koldewey HJ, MIllner-Gulland EJ. The interaction between seaweed farming as an alternative occupation and fisher numbers in the Central Philippines. Conservation Biology. 2011;26(2):324-34.

40. Jeppesen E, Søndergaard M, Mortensen E, Kristensen P, Riemann B, Jensen HJ, et al. Fish manipulation as a lake restoration tool in shallow, eutrophic temperate lakes 1: cross-analysis of three Danish case-studies. Hydrobiologia. 1990;200/201:205-18.

____________________________________________________________________________________________________________________________

This additional file is part of the article *Principles and framework for assessing the risk of bias for studies included in comparative quantitative environmental systematic reviews.* Environmental Evidence journal 2022.
