# Supplementary material for: Principles and framework for assessing the risk of bias for studies included in comparative quantitative environmental systematic reviews
Source: Environ Evid. Author manuscript; Available in PMC 2024 Jan 23. (PMC10805236; doi:10.1186/s13750-022-00264-0)
Supplement: s4 — Additional file 4. Examples of risk of bias tools and checklists for specific study designs. [file NIHMS1948588-supplement-s4.docx]

**Additional file 4 Examples of risk of bias tools and checklists for specific study designs**

| **Study design** | **Tools and checklists** | **Sources of bias covered in tool or checklist** |
| --- | --- | --- |
| **Randomised controlled trials** | Cochrane risk of bias tool (1) | ● Selection bias (selection & allocation of participants)  ● Performance bias (deviations from intended interventions)  ● Detection bias (measurement of the outcome)  ● Attrition bias (missing outcome data)  ● Reporting bias (selection of the outcome and/or reported result) |
|  | Cochrane RoB2 tool (2) | ● The randomisation process  ● Baseline confounding  ● Deviations from intended interventions  ● Measurement of the outcome  ● Missing outcome data  ● Selection of the reported result |
|  | Cochrane EPOC checklist (3) | ● Random sequence generation  ● Allocation concealment  ● Baseline outcome measurements similar  ● Baseline characteristics similar  ● Incomplete outcome data  ● Knowledge of the allocated interventions adequately prevented during the study  ● Protection against contamination  ● Selective outcome reporting |
|  | NIH tool for controlled intervention studies (4) | ● Baseline similarity of groups  ● Random sequence generation  ● Allocation concealment  ● Participant and investigator blinding  ● Outcome assessor blinding  ● Dropout (including intention to treat and crossover)  ● Confounding with other interventions  ● Outcome measures assessment |
| **Randomised and non-randomised comparative environmental studies** | CEE critical appraisal tool (prototype) (5) | ● Confounding  ● Post-intervention/selection biases  ● Misclassified comparison biases in observational  studies (deviation from  exposure)  ● Performance biases in experimental studies  (deviation from intervention)  ● Detection biases  ● Outcome reporting biases  ● Outcome assessment biases (biases in statistical  methods) |
| **Non-randomised studies of interventions** | Cochrane ROBINS-I tool (6) | ● Confounding  ● Selection of participants  ● Deviations from intended interventions  (includes performance bias in experimental studies)  ● Measurement of the outcome  ● Missing outcome data  ● Selection of the reported result |
| **Human experi-mental study** | OHAT (2015, 2019) (7, 8) | ● Selection bias  ● Performance bias  ● Attrition/Exclusion bias  ● Detection bias  ● Selective reporting bias |
| **Controlled before-after studies** | Cochrane EPOC checklist (3) | Same criteria as Cochrane EPOC criteria above for randomised controlled trials |
| **Non-randomised trials** | Cochrane EPOC checklist (3) | Same criteria as Cochrane EPOC criteria above for randomised controlled trials |
| **Non-randomised experimental studies** | JBI checklist for quasi-experimental studies (9) | ● Selection of groups  ● Measurement of the outcome  ● Missing outcome data  ● Statistical analysis |
| **Non-randomised studies (general)** | Wells et al. (2013) (10) | ● Selection of the outcome and/or reported result  ● Selective analysis reporting  ● Confounding |
| **Experimental animal studies** | Hooijmans et al. (2014) (11)  OHAT (2015, 2019) (7, 8)  Rooney et al. (2014) (12)  Krauth et al. (2013) (13) | ● Selection of groups  ● Deviations from intended interventions / exposures  ● Measurement of the outcome  ● Missing outcome data  ● Selection of the outcome and/or reported result  ● Confounding |
| **Diagnostic test accuracy studies** | QUADAS (14) and QUADAS-2 (15) tools | ● Specific named biases inherent in test accuracy studies |
| **Behavioural ecological studies** | Stuber et al. (2013) (16) | ● Measurement of the outcome (NB this is an example, not a checklist) |
| **Interrupted time series** | Greenhalgh et al. (2005) (17) | ● Secular changes (i.e. confounding trends)  ● Measurement of the outcome  ● Missing outcome data |
|  | Cochrane EPOC checklist (3) | ● Intervention independent of other changes  ● Shape of the intervention effect pre-specified  ● Intervention unlikely to affect data collection  ● Knowledge of the allocated interventions adequately prevented during the study  ● Incomplete outcome data adequately accounted for  ● Selective outcome reporting |
| **Case control studies** | Viswanathan et al. (2013) (18)  OHAT (2015, 2019) (7, 8)  CASP checklist (19)  JBI checklist (20)  NIH tool for case control studies (4) | ● Selection of groups  ● Measurement of the exposure  ● Measurement of the outcome  ● Exposure/risk measure implementation  ● Exposure/risk assessor blinding  ● Confounding  ● Statistical analysis |
| **Cohort studies** | Viswanathan et al. (2013) (18)  OHAT (2015, 2019) (7, 8)  Jarde et al. (2013) (21)  CASP checklist (22)  JBI checklist (23) | ● Selection of groups  ● Measurement of the outcome  ● Missing outcome data  ● Confounding  ● Statistical analysis |
| **Observational cohort and cross-sectional studies** | NIH tool for observational cohort and cross-sectional studies (4) | ● Similarity of source populations  ● Exposures measured prior to outcomes  ● Sufficient timeframe to detect an association  ● Appropriate levels of exposure  ● Exposure assessed more than once over time  ● Blinding of outcome assessors  ● Missing data  ● Key potential confounding variables |
| **Cross-sectional studies** | Viswanathan et al. (2013) (18)  OHAT (2015, 2019) (7, 8) | ● Selection of groups  ● Measurement of the outcome  ● Confounding |
| **Questionnaires or surveys** | Choi & Pak (2005) (24)  Sedgwick (2013) (25) | ● Selection of participants  ● (Non-) response  ● Recall  ● Missing outcome data  ● Other sources of bias inherent in surveys |
| **Case series** | Viswanathan et al. (2013) (18)  OHAT (2015, 2019) (7, 8)  JBI checklist (26)  NIH tool for case series (4) | ● Selection of cases  ● Comparability of cases  ● Measurement of the outcome  ● Adequacy of follow-up  ● Confounding  ● Statistical analysis |
| **Prevalence studies** | JBI checklist (27) | ● Selection of participants  ● Measurement of the exposure  ● Statistical analysis |
| **Case reports** | JBI checklist (28) | Bias is difficult to assess; focus on likely plausibility, clarity, applicability of the reported result |
| Note that some tools include other constructs besides risk of bias (internal validity), e.g. precision, or clarity of reporting. Only the internal validity items are summarised here. | | |

**References**

1. Higgins J, Altman D, Gøtzsche P, Jüni P, Moher D, Oxman A, et al. The Cochrane Collaboration's tool for assessing risk of bias in randomised trials. BMJ. 2011;343:d5928:1-9.

2. Higgins JPT, Savović J, Page MJ, Sterne JAC, Hróbjartsson A, Boutron I, et al. Revised Cochrane risk-of-bias tool for randomized trials (RoB 2). Cochrane Methods Goup; 2019.

3. Cochrane Effective Practice and Organisation of Care (EPOC). Suggested risk of bias criteria for EPOC reviews 2017.

4. National Institutes of Health (NIH). Study quality assessment tools 2014 [Available from: <https://www.nhlbi.nih.gov/health-topics/study-quality-assessment-tools>].

5. CEE (Collaboration for Environmental Evidence). Collaboration for Environmental Evidence Critical Appraisal Tool Version 0.3 (Prototype) 2021 [Available from: <https://environmentalevidence.org/cee-critical-appraisal-tool/>].

6. Sterne J, Hernán M, Reeves B, Savovíc J, Berkman N, Viswanathan M, et al. ROBINS-I: a tool for assessing risk of bias in non-randomised studies of interventions. BMJ. 2016;355:i4919:1-7.

7. National Toxicology Program US Department of Health and Human Services. Handbook for Conducting a Literature-Based Health Assessment Using OHAT Approach for Systematic Review and Evidence Integration. Office of Health Assessment and Translation (OHAT), Division of the National Toxicology Program, National Institute of Environmental Health Sciences; 2019.

8. OHAT (Office of Health Assessment and Translation). Handbook for conducting a literature-based health assessment using OHAT approach for systematic review and evidence integration. 2015.

9. Joanna Briggs Institute. Checklist for quasi-experimental studies (non-randomised experimental studies). 2017.

10. Wells GA, Shea B, Higgins JPT, Sterne J, Tugwell P, Reeves BC. Checklists of methodological issues for review authors to consider when including non-randomized studies in systematic reviews. Research Synthesis Methods. 2013;4:63-77.

11. Hooijmans C, Rovers M, de Vries R, Leenaars M, Ritskes-Hoitinga M, Langendam M. SYRCLE's risk of bias tool for animal studies. BMC Medical Research Methodology. 2014;14:43:1-9.

12. Rooney AA, Boyles AL, Wolfe MS, Bucher JR, Thayer KA. Systematic review and evidence integration for literature-based environmental health science assessments. Environmental Health Perspectives. 2014;122(7):711-8.

13. Krauth D, Woodruff T, Bero L. Instruments for assessing risk of bias and other methodological criteria of published animal studies: a systematic review. Environmental Health Perspectives. 2013;121:985-92.

14. Whiting P, Weswood M, Rutjes A, Reitsma J, Bossuyt P, Kleijnen J. Evaluation of QUADAS, a tool for the quality assessment of diagnostic accuracy studies. BMC Med Res Methodol. 2006;6:9:1-8.

15. Whiting P, Rutjes A, Westwood M, Mallett S, Deeks J, Reitsma J, et al. QUADAS-2: a revised tool for the quality assessment of diagnostic accuracy studies. Ann Intern Med. 2011;155:529-36.

16. Stuber E, Araya-Ajoy Y, Mathot K, Mutzel A, Nicolaus M, Wijmenga J, et al. Slow explorers take less risk: a problem of sampling bias in ecological studies. Behavioural Ecology. 2013;24:1092-8.

17. Greenhalgh T, Robert G, Bate P, Macfarlane F, Kyriakidou O. Diffusion of innovations in health service organisations: A systematic literature review. Appendix 2: Critical appraisal checklists. 2005.

18. Viswanathan M, Berkman N, Dryden D, Hartling L. Assessing risk of bias and confounding in observational studies of interventions or exposures: further development of the RTI Item Bank. Rockville, MD, USA; 2013.

19. CASP (Critical Appraisal Skills Programme). CASP case control study checklist. 2018.

20. Joanna Briggs Institute. Checklist for case control studies. 2017.

21. Jarde A, Losilla J-M, Vives J, Rodrigo M. Q-Coh: A tool to screen the methodological quality of cohort studies in systematic reviews and meta-analyses. International Journal of Clinical and Health Psychology. 2013;13:138-46.

22. CASP (Critical Appraisal Skills Programme). CASP cohort study checklist. 2018.

23. Joanna Briggs Institute. Checklist for cohort studies. 2017.

24. Choi BCK, Pak AWP. A catalog of biases in questionnaires. Preventing Chronic Disease. 2005;2 (1), A13:1-13.

25. Sedgwick P. Questionnaire surveys: sources of bias. BMJ. 2013;347:f5265:1-2.

26. Joanna Briggs Institute. Checklist for case series. 2017.

27. Joanna Briggs Institute. Checklist for prevalence studies. 2017.

28. Joanna Briggs Institute. Checklist for case reports. 2017.

_________________________________________________________________________________

This additional file is part of the article *Principles and framework for assessing the risk of bias for studies included in comparative quantitative environmental systematic reviews.* Environmental Evidence journal 2022.
