# Supplementary material for: Principles and framework for assessing the risk of bias for studies included in comparative quantitative environmental systematic reviews
Source: Environ Evid. Author manuscript; Available in PMC 2024 Jan 23. (PMC10805236; doi:10.1186/s13750-022-00264-0)
Supplement: s5 — Additional file 5. General criteria for considering risks of bias in comparative quantitative environmental research studies. [file NIHMS1948588-supplement-s5.docx]

**Additional file 5 General criteria for considering risks of bias in comparative quantitative environmental research studies**

Note: This additional file is not intended for use as a risk of bias tool. It provides a guide to the classes of bias that review teams should consider and may be useful to help determine whether risk of bias tools that are selected (or developed) by review teams capture all relevant sources of bias.

| **1. Risk of bias due to confounding** | **Study characteristics that increase internal validity (reduce risk of bias)** | **Study characteristics that reduce internal validity (increase risk of bias)** |
| --- | --- | --- |
| **General question: Apart from the intended exposure, could any baseline factors be associated with both the exposure and outcome of interest? (no=low risk of bias)**  **Rationale:** Given the complexity of ecological systems, care should be taken to ensure that all possible biotic and abiotic confounding variables have been identified and accounted for. Appropriate methods to control for confounders may include stratification, regression, matching, propensity scores, standardisation, and inverse probability weighting. However, each method depends on an assumption that there is no unmeasured or residual confounding (1). | ● No important confounding variables that could explain differences in outcomes between exposure and comparator groups were likely to be present (based on a thorough consideration of the study system).  *If any important confounding variables were present:*  ● The confounders were controlled by using randomisation (see considerations relating to randomisation under selection bias below).  ● The confounders were controlled by using an appropriate statistical analysis method, and the values of confounding variables used in the analysis were measured validly and reliably  ● After being controlled for, all potential confounders were well-balanced between study groups. | ● Important potential confounding variables were present and were either not controlled for appropriately in the study design (i.e. not controlled for at all, or controlled for using an inappropriate method).  ● Differences in outcomes among sites or samples could be explained by one or more known confounding factors.  ● Confounding variables were incorrectly identified (i.e. variables that were not associated with both the exposure and outcome of interest were adjusted for in the analysis). |

| **2. Risk of bias in selection of study sites, study groups or participants (selection bias)** | **Study characteristics that increase internal validity (reduce risk of bias)** | **Study characteristics that reduce internal validity (increase risk of bias)** |
| --- | --- | --- |
| **General question: Were all study groups balanced on all important variables that could influence the outcome of interest? (yes=low risk of bias)**  **Rationale:** Unless all (i.e. both measured and unmeasured) variables other than the intended exposure are balanced across all study sites and treatment groups then there is a risk that the observed outcome of interest might not be explained solely by the exposure of interest, potentially leading to under- or over-estimation of the true outcome value and undermining the ability of a study to demonstrate causality. Randomisation of group allocations is the only approach that, if conducted appropriately, can definitively ensure that both measured and unmeasured variables are likely to be balanced across the study groups. In observational studies, where randomisation is not feasible an attempt should be made to ensure that study sites and treatment groups are well-matched for all known factors, and any baseline imbalances should be accounted for in analyses. In such cases an assumption has to be made that unknown and unmeasured factors are either well-balanced across sites and groups, or are unimportant. Review teams should consider whether this assumption is reasonable in the context of each individual study. | *Where randomisation is feasible:*  ● Experimental studies: Random selection of exposure and comparator sites and random assignment of the treatments or participants (e.g. animal or human) to exposure groups.  ● Observational studies: Study sites were selected at random, or randomly within specific parameters of interest (e.g. stratified by soil type or land use history).  *Where randomisation is not feasible:*  ● An appropriate method was used to ensure that all potential explanatory factors were balanced across study sites and treatment groups.  ● Any baseline imbalances in potential explanatory factors were accounted for in analyses.  *Where randomisation is claimed by study authors:*  ● An appropriate method was used to generate a random allocation sequence.  ● The allocation sequence was concealed from study investigators until sites/exposures/comparators had been assigned.  ● There is evidence that randomisation was successful (e.g. group characteristics are well-balanced). | *Where randomisation is feasible:*  ● Study site selection was not random.  ● Assignment of treatments to sites was not random.  ● Assignment of participants to study groups was not random.  *Where randomisation is not feasible:*  ● An inadequate attempt was made to ensure that all known potential explanatory factors were balanced across study sites and treatment groups (e.g. no attempt at matching, or groups were poorly-matched).  ● Imbalances in known potential explanatory factors were not adequately accounted for in analyses.  *Where randomisation is claimed by study authors:*  ● The method of allocation of sites/treatments was not random.  ● The allocation sequence could have been manipulated by investigators, because it was not adequately concealed.  ● Baseline differences in study sites or treatment group characteristics suggest a problem with the randomisation process. |

| **3. Risk of bias due to misclassification of the exposure or comparator - observational studies only** | **Study characteristics that increase internal validity (reduce risk of bias)** | **Study characteristics that reduce internal validity (increase risk of bias)** |
| --- | --- | --- |
| **General question: Were the exposure and comparator clearly defined and adhered to as intended? (yes=low risk of bias)**  **Rationale:** Clear definition of the exposure is essential to ensure that the treatment effect cannot be explained by variation in whether the intended exposure was adhered to. For example, if an exposure consists of multiple chemicals there may be a possibility that the populations or study areas of interest are not exposed to all of the intended chemicals. Subject knowledge is important to ensure that complex exposures (e.g. involving chemical mixtures, or multicomponent behavioural activities or processes) are understood well enough to be clearly definable and match the exposure that is required by the review question. | ● The intended exposure was precisely defined and there is no evidence for any deviations from the intended exposure. | ● The intended exposure was defined imprecisely or is unclear.  ● There were deviations from the intended exposure, e.g. the populations or study areas were not exposed to all components of the exposure. |

| **4. Risk of bias due to misclassification of the exposure or comparator (performance bias) – experimental studies only** | **Study characteristics that increase internal validity (reduce risk of bias)** | **Study characteristics that reduce internal validity (increase risk of bias)** |
| --- | --- | --- |
| **General question: Were there any important reasons why the intended exposure or comparator were not implemented, or received by participants, as intended? (no=low risk of bias)**  **Rationale:** Important deviations from intended exposures may lead to under- or over-estimation of the outcome of interest. Deviations could occur if the intensity of exposure (e.g. concentration of a chemical) differs from that intended, or differs between treatment replicates, or if not all of the intended target organisms or study participants are exposed for the full duration of the intended exposure period. If study investigators, or study participants, are aware of which study groups received the exposure and comparator, then they may be able to manipulate how an exposure is implemented (often referred to as performance bias). For multi-component interventions all of the individual components should be implemented as intended, across all replicates. If any relevant co-interventions could occur these should be balanced across all study groups. | ● Study investigators and, where relevant, participants, were unaware of the exposure and comparator study group identity (i.e. they were masked/blinded).  ● There is evidence that the exposure was implemented successfully, as intended (i.e. there were no deviations from the intended exposure, or any deviations were balanced between all study groups).  ● If any important additional co-exposures or co-interventions were present these were balanced across all study groups. | ● Awareness of the treatment group allocations by study investigators or participants, who could have influenced how the exposure was delivered.  ● There were important deviations from the intended exposure which were unbalanced across the study groups.  ● The exposure was not implemented as intended, or not all participants received their intended treatment.  ● Important additional co-exposures or co-interventions were present and were unbalanced across the study groups. |

| **5. Risk of bias due to missing data (attrition bias)** | **Study characteristics that increase internal validity (reduce risk of bias)** | **Study characteristics that reduce internal validity (increase risk of bias)** |
| --- | --- | --- |
| **General question: Were there any systematic differences between study groups in the number of missing data, the values of the missing data for the outcome of interest, or the reasons for the data being missing? (no=low risk of bias)**  **Rationale:** Missing outcome data in a study can lead to errors in estimation of the outcome of interest, particularly if the number of missing data is large, unbalanced across study groups or across outcome assessment time points, or the reasons for data being missing are related to the exposure and/or outcome. Missing data could arise if, for example, participants drop out of a study, samples are not collected as intended, or samples are damaged or lost after collection. Statistical methods may be able to correct for missing data under some circumstances (1).  Note that bias due to missing data is a type of selection bias (see Table 2 in the paper) | ● There were no missing data  *If there were any missing data:*  ● The proportion missing was too small to affect the effect estimate of interest.  ● The reasons for data being missing did not differ systematically between the study groups.  ● The reasons for data being missing were not related to the exposure or the outcome of interest.  ● The true values of the missing data were unlikely to differ systematically from the values of the non-missing data.  ● There is evidence that results were robust to the presence of missing data (i.e. missing data were appropriately accounted for in the analysis of the outcome of interest). | ● The number of missing data was sufficient to have potentially influenced the treatment effect estimate.  ● There was a systematic difference between exposure and comparator groups or between assessment times in the number of missing data and/or the reasons for data being missing.  ● The reasons for data being missing were related to the exposure group and/or outcome of interest.  ● The true values of the missing data were likely to differ systematically from the values of the non-missing data.  ● Missing data were not corrected for appropriately in the analysis (i.e. not corrected for at all, or corrected for using an inappropriate method). |

| **6. Risk of bias in measurement of the outcome (detection bias)** | **Study characteristics that increase internal validity (reduce risk of bias)** | **Study characteristics that reduce internal validity (increase risk of bias)** |
| --- | --- | --- |
| **General question: Was an appropriate method used to measure the outcome across all study groups? (yes=low risk of bias)**  **Rationale:** Systematic errors in the measurement of outcomes may occur if the method of outcome measurement is inappropriate, or if it differs between study groups. Differences in outcome measurement between study groups could occur for various reasons, for example if the outcome assessors differ between the study groups and also differ in their experience of using the outcome measurement technique; or if data collection equipment differs (or is calibrated differently) between intervention and comparator groups. Outcomes that require judgement, such as self-reported questionnaire responses, are particularly prone to bias. Where possible, outcome assessors should be masked/blinded to ensure that they are not aware of the identity of the study groups when measuring subjective outcomes. | ● An appropriate method was used to measure the outcome of interest AND the same method of measuring the outcome was applied to all study groups AND investigators who measured outcomes were not aware of the identity of the study groups because they were masked/ blinded to the exposure/ intervention received.  ● Outcome assessors were aware of the identity of study groups but it is unlikely that they could have systematically influenced the outcome measurement (e.g. if the outcome was measured automatically or not subjective). | ● An inappropriate method was used for measuring outcomes.  ● The method for measuring outcomes differed systematically between the study groups, sampling times, or locations.  ● Investigators or participants measuring outcomes were aware of the identity of the study groups and might have (intentionally or unintentionally) introduced systematic differences between study groups in the way outcomes were measured (e.g. self-report questionnaire responses could be influenced by knowledge of exposure group allocation). |

| **7. Risk of bias in selection of the reported result (outcome reporting bias)** | **Study characteristics that increase internal validity (reduce risk of bias)** | **Study characteristics that reduce internal validity (increase risk of bias)** |
| --- | --- | --- |
| **General question: Were all outcome measurements reported appropriately? (yes=low risk of bias)**  **Rationale:** Systematic error in estimation of the outcome may occur if study authors report only some of the available outcome measurements that were made (e.g. selecting from multiple measurements, time points or analysis methods). Note that selective reporting may involve manipulation of the outcome, such as converting a continuously-scaled outcome to categorical data with arbitrary cut-offs (1). | ● There is no evidence that any of the available measurements for the outcome of interest have been selectively excluded by the study authors based on their direction, magnitude, and/or statistical significance (i.e. no evidence of selection of specific measurements, analysis methods, or time points). | ● There is evidence for selective reporting of the outcome measurements, based on their direction, magnitude, and/or statistical significance.  ● Outcomes are not reported for all of the time points assessed.  ● Outcomes reported do not reflect the full data collected (e.g. continuous data were collected but are reported as categorical data with arbitrary cut-offs). |

| **8. Risk of bias due to an inappropriate statistical analysis approach (statistical conclusion validity)** | **Study characteristics that increase internal validity (reduce risk of bias)** | **Study characteristics that reduce internal validity (increase risk of bias)** |
| --- | --- | --- |
| **General question: Could the method of statistical analysis have introduced bias in the reported result for the outcome of interest? (no=low risk of bias)**  **Rationale:** Statistical analyses may be complex, are sometimes based on strong assumptions, and may not always be implemented appropriately or reported clearly. It is therefore important to consider whether the statistical analysis conducted in a research study could have introduced systematic error in the effect estimate. Possible problems include incorrect specification of the statistical model, errors in the input data, implausibility of the statistical assumptions, and unit of analysis issues such as non-independent data analysed as though they were independent. A “face validity” check on the results of statistical analyses should always be performed, i.e. a check that the results are plausible and realistic. | ● All steps of the statistical calculations are logical, transparently reported, and free from systematic errors.  ● Any assumptions are appropriate and transparently reported.  ● Statistical calculations and assumptions are applied consistently to all study groups.  ● There is evidence of the face validity of the analysis (i.e. the results are plausible and consistent with the input data).  ● Tests of analysis results are provided where appropriate (e.g. sensitivity analyses) which demonstrate robustness of the analysis. | ● Correlations are not appropriately accounted for (e.g. non-independent data such as repeated within-subject observations analysed as independent).  ● Presence of pseudoreplication (2) (pseudoreplication is relatively common (2, 3)).  ● Violation of statistical assumptions in such a way that systematic error could be introduced.  ● Statistical model based on implausible assumptions about the environmental system.  ● Inappropriate units of analysis or output scales.  ● Bias or other errors in the input data.  ● Inappropriate extrapolation (e.g. based on inadequate sample size or unrepresentative source data).  ● Implausible results of statistical analysis. |

| **9. Other risks of bias** | **Study characteristics that increase internal validity (reduce risk of bias)** | **Study characteristics that reduce internal validity (increase risk of bias)** |
| --- | --- | --- |
| **General question: Were there any other sources of potential bias additional to those in the eight classes of bias listed above? (no=low risk of bias)**  **Rationale:** Issues of replication, sample size, and temporal and spatial scaling of studies may be sources of systematic error. It is important to consider the impact on the treatment effect estimate of interest, e.g. would any aspects of the study design introduce systematic error in the treatment effect estimates beyond those already accounted for in the bias classes above? Some aspects, such as pseudoreplication, could be considered an aspect of statistical conclusion validity (see above). The review team should provide a rationale for any thresholds used, such as the minimum number of replicates or minimum sample size required where these could influence internal validity, based on quantitative evidence where possible. Depending on the study designs of interest, some sources of bias may be captured in specific tools that describe additional classes of bias (domains) to those listed above (e.g. studies evaluating test accuracy (4, 5)). | ● No further sources of potential bias beyond those in the eight classes of bias listed above were identified. | ● Further potential sources of bias were present which are not covered in the eight classes of bias listed above. |

**References**

1. Sterne JAC, Higgins JPT, Elbers RG, Reeves BC, ROBINS-I Development Group. Risk of bias in non-randomized studies of interventions (ROBINS-I): detailed guidance. 2016.

2. Hurlbert SH. Pseudoreplication and the design of ecological field experiments. Ecological Monographs. 1984;54(2):187-211.

3. Taylor JJ, Rytwinski T, Bennett JR, Smokorowski KE, Lapointe NWR, Janusz R, et al. The effectiveness of spawning habitat creation or enhancement for substrate‑spawning temperate fish: a systematic review. Environmental Evidence. 2019;8:19:1-31.

4. Whiting P, Rutjes A, Westwood M, Mallett S, Deeks J, Reitsma J, et al. QUADAS-2: a revised tool for the quality assessment of diagnostic accuracy studies. Ann Intern Med. 2011;155:529-36.

5. Whiting P, Weswood M, Rutjes A, Reitsma J, Bossuyt P, Kleijnen J. Evaluation of QUADAS, a tool for the quality assessment of diagnostic accuracy studies. BMC Med Res Methodol. 2006;6:9:1-8.

____________________________________________________________________________________________________________________________

This additional file is part of the article *Principles and framework for assessing the risk of bias for studies included in comparative quantitative environmental systematic reviews.* Environmental Evidence journal 2022.
