# Supplementary material for: Principles and framework for assessing the risk of bias for studies included in comparative quantitative environmental systematic reviews
Source: Environ Evid. Author manuscript; Available in PMC 2024 Jan 23. (PMC10805236; doi:10.1186/s13750-022-00264-0)
Supplement: s6 — Additional file 6. Examples of critical appraisal instruments assessed for fitness for purpose against the FEAT principles. [file NIHMS1948588-supplement-s6.docx]

**Additional file 6 Examples of critical appriasal instruments assessed for fitness-for-purpose against the FEAT principles**

| **FEAT principles as applicable to internal validity** | | **Examples of risk of bias tools assessed against the FEAT principles** | | |
| --- | --- | --- | --- | --- |
| ***Principle*** | ***Explanation*** | **Newcastle Ottawa Scale**  **[1]** | **Joanna Briggs Institute critical appraisal tool for cohort studies** **[2]** | **OHAT Risk of Bias tool**  **[3]** |
| **FOCUSED** | Critical appraisal should be directed at the internal validity of the included studies, assessed as risk of bias. | Does target internal validity, the potential for systematic error in the results of case-control and cohort studies. | Does target internal validity, the potential for systematic error in the results of a cohort study. | Does target internal validity, the potential for systematic error in the results of human controlled trials, experimental animal studies, cohort studies, case-control studies, cross-sectional studies and case series. |
| **EXTENSIVE** | For a review that includes randomised studies, key elements of study design which threaten internal validity include random allocation of subjects to exposure and control arms, blinding of investigators, and selective reporting of results. | Does not cover all of the important threats to the internal validity of a study. For example, selective reporting is not assessed. | Does not cover all of the important threats to the internal validity of a cohort study. For example, selective reporting and blinding of investigators (an aspect of performance bias) is not assessed. | Covers all of the important threats to the internal validity of a study. Explains why financial conflicts of interest are not an explicit domain and indicates how they should be handled. |
| **APPLIED** | Reviewers should use a prespecified appraisal tool to make qualitative judgements about risk of bias for each domain of internal validity, unless quantitative judgements can be supported by good empirical evidence. Reviewers should be trained in use of the tool, with judgements made in duplicate. | Is a checklist of tightly-defined criteria. Equivalent methods to those prescribed are inappropriately downgraded. NOS also treats each shortcoming as equal regardless of whether e.g. lack of blinding might pose a greater threat to internal validity than differences in response rate to exposure surveys. The final rating is a total score, which can conceal from analysis important differences between studies. | Is a checklist of criteria but is not intended to generate a validity classification. Does not consistently differentiate whether an element of study design was merely implemented or was implemented in a way which reduces threats to internal validity. It produces a series of yes/no/unclear answers to the checklist, but it is not clear how these answers should be interpreted when it comes to their impact on the internal validity of a cohort study. | Presents a series of questions about approaches taken to minimise risk of bias in each appraisal domain, eliciting judgements of high / probably high / probably low / low risk of bias. Based on judgements for key bias domains, studies are assigned to one of three tiers of internal validity. The tiers are used for subgroup analysis and are incorporated into a GRADE-based approach to determining certainty in results of the systematic review. |
| **TRANSPARENT** | Reviewers should record in an appraisal report for every included study their reasoning for each risk of bias judgement, quoting as justification relevant text from the study documentation. | Does not ask for reviewers to give reasons for their judgements. | Does not ask for reviewers to give reasons for their judgements. | Asks reviewers to give reasons for their judgements and provide brief quotations from the text to support them. |

**References**

1. Wells G, Shea B, O'Connell D, Peterson J, Welch V, Losos M, et al. The Newcastle-Ottawa scale (NOS) for assessing the quality of nonrandomised studies in meta-analyses. Ottawa, Canada: University of Ottawa, 2009.

2. Joanna Briggs Institute. Checklist for cohort studies. 2017.

3. OHAT (Office of Health Assessment and Translation). Handbook for conducting a literature-based health assessment using OHAT approach for systematic review and evidence integration. 2015.

__________________________________________________________________________________________________________________________________________________________________________________________________________________________________________________________________________________________________________

This additional file is part of the article *Principles and framework for assessing the risk of bias for studies included in comparative quantitative environmental systematic reviews.* Environmental Evidence journal 2022.
