# Supplementary material for: Principles and framework for assessing the risk of bias for studies included in comparative quantitative environmental systematic reviews
Source: Environ Evid. Author manuscript; Available in PMC 2024 Jan 23. (PMC10805236; doi:10.1186/s13750-022-00264-0)
Supplement: s7 — Additional file 7. Checklist for planning a risk of bias assessment. [file NIHMS1948588-supplement-s7.docx]

**Additional file 7 Checklist for planning a risk of bias assessment**

| **FOCUS**  *The assessment should be appropriately FOCUSED* | ● The focus should be on internal validity (i.e. risk of bias)  ● If other “quality” constructs are assessed these should be separable from internal validity. For rationale see section *“Internal validity in relation to other study quality constructs”* in the paper.  ● Consider any implications of (lack of) blinding/masking when assessing risks of bias (Box 2 in the paper). |
| --- | --- |
| **EXTENT**  *The assessment should be EXTENSIVE* | ● All important threats to internal validity (i.e. all domains of potential bias) that are relevant to the included study designs should be included (for the key domains of bias that should be assessed see section *“Types of bias and appraisal tools relevant to environmental studies”* and Table 2 in the paper)  ● A clear rationale should be provided for the inclusion or exclusion of each bias domain (e.g. a robust stakeholder-informed decision making process to ensure that all confounders have been identified and/or use of an existing risk of bias instrument). If an existing instrument is used, evidence should be provided that it is fit for purpose. For examples of assessing fitness for purpose of existing instruments see Additional file 6. |
| **APPLICATION**  *The assessment results should be APPLIED* | ● The output of critical appraisal should be appropriately structured, i.e. with clearly defined validity classes that are correctly interpreted and applied (see section *“Develop a new risk of bias tool”* and Table 4 in the paper) and a logical process for determining the overall validity (risk of bias) for each outcome (Table 5 in the paper).  ● Avoid using numerical scores that conflate quality constructs or imply that qualitative constructs have equal weight (see Box 3 in the paper).  ● The critical appraisal output should inform the data synthesis for each outcome included in the review, for both quantitative and narrative data syntheses, where conducted (see section *“Apply”* and Table 7 in the paper). |
| **TRANSPARENCY**  *The assessment should be fully TRANSPARENT* | ● The rationale for each judgement in the risk of bias assessment should be clearly documented for each outcome in each study (see section *“Report”* and Table 8 in the paper)  ● The methods and results of all steps of the critical appraisal process should be clearly reported. For examples of transparency issues in CEE reviews see Additional file 2. |
| **PROCESS**  *Key aspects of the methods required to mitigate against the review team introducing bias and other errors* | ● The critical appraisal should be based on the systematic review protocol, with any deviations clearly justified  ● Assessments should be conducted by at least 2 reviewers, with clear documentation of any disagreements and how these were resolved (see Table 6 in the paper)  ● The topic expertise composition and affiliations of the review team and any stakeholders advising the assessment should be specified and all participants in the review should be free from conflicts; authors or other contributors to studies included in the review should not critically appraise their own studies (see section *“Prepare the team”* in the paper) |

_________________________________________________________________________________

This additional file is part of the article *Principles and framework for assessing the risk of bias for studies included in comparative quantitative environmental systematic reviews.* Environmental Evidence journal 2022.
