# Supplementary material for: Principles and framework for assessing the risk of bias for studies included in comparative quantitative environmental systematic reviews
Source: Environ Evid. Author manuscript; Available in PMC 2024 Jan 23. (PMC10805236; doi:10.1186/s13750-022-00264-0)
Supplement: s8 — Additional file 8. Checklist for critiquing a risk of bias assessment. [file NIHMS1948588-supplement-s8.docx]

**Additional file 8 Checklist for critiquing a risk of bias assessment**

| **FOCUSED?** | ● Does the critical appraisal focus on internal validity (i.e. risk of bias)? | Yes/No |
| --- | --- | --- |
|  | ● If there are other constructs assessed (e.g. external validity) are these separable from internal validity? | Yes/no |
| **EXTENSIVE?** | ● Are all important threats to internal validity (i.e. all sources of potential bias) that are relevant to the included study designs assessed? | Yes/No |
| **APPLIED?** | ● Is the output of the critical appraisal appropriately structured, i.e. clearly defined validity classes, with a logical process for determining the overall validity (risk of bias) for each outcome? | Yes/No |
|  | ● Have each of the criteria for assessing risk of bias been correctly interpreted and applied? | Yes/No |
|  | ● Does the critical appraisal output inform the data synthesis for each outcome included in the review (applies to both quantitative and narrative syntheses, where conducted)? | Yes/No |
| **TRANSPARENT?** | ● Are the reasons for coming to each judgement in the risk of bias assessment sufficiently clearly documented? | Yes/No |
| **KEY PROCESS ELEMENTS** | ● Is the critical appraisal process free from potential sources of bias or other errors, i.e. the methods followed the protocol, at least two reviewers conducted each assessment, reviewers did not assess their own articles, and no conflicts of interest were evident? | Yes/No |

_________________________________________________________________________________

This additional file is part of the article *Principles and framework for assessing the risk of bias for studies included in comparative quantitative environmental systematic reviews.* Environmental Evidence journal 2022.
